# Mapping the cooperativity pathways in spin crossover complexes†

**DOI:** 10.1039/d0sc05819j

**Published:** 2020-11-16

**Authors:** Matthew G. Reeves, Elodie Tailleur, Peter A. Wood, Mathieu Marchivie, Guillaume Chastanet, Philippe Guionneau, Simon Parsons

**Affiliations:** Centre for Science at Extreme Conditions, EaStCHEM School of Chemistry, The University of Edinburgh King's Buildings, West Mains Road Edinburgh Scotland EH9 3FJ UK S.Parsons@ed.ac.uk; CNRS, Univ. Bordeaux, Bordeaux INP, ICMCB, UMR 5026 87 av. Dr A. Schweitzer F-33600 Pessac France Mathieu.Marchivie@icmcb.cnrs.fr Philippe.Guionneau@icmcb.cnrs.fr; Cambridge Crystallographic Data Centre 12 Union Road Cambridge England CB2 1EZ UK wood@ccdc.cam.ac.uk

## Abstract

Crystal packing energy calculations are applied to the [Fe(PM-L)_2_(NCS)_2_] family of spin crossover (SCO) complexes (PM-L = 4-substituted derivatives of the *N*-(2-pyridylmethylene)-4-aminobiphenyl ligand) with the aim of relating quantitatively the cooperativity of observed SCO transitions to intermolecular interactions in the crystal structures. This approach reveals a linear variation of the transition abruptness with the sum of the magnitudes of the interaction energy changes within the first molecular coordination sphere in the crystal structure. Abrupt transitions are associated with the presence of significant stabilising and destabilising changes in intermolecular interaction energies. While the numerical trend established for the PM-L family does not directly extend to other classes of SCO complex in which the intermolecular interactions may be very different, a plot of transition abruptness against the range of interaction energy changes normalised by the largest change shows a clustering of complexes with similar transition abruptness. The changes in intermolecular interactions are conveniently visualised using energy difference frameworks, which illustrate the cooperativity pathways of an SCO transition.

## Abstract 2

The abruptness of spin crossover (SCO) is related to intermolecular energy changes occurring over the course of an SCO transition. Crossover is abrupt when SCO-induced strain is accommodated synergistically in a few key interactions.

## Graphical abstract

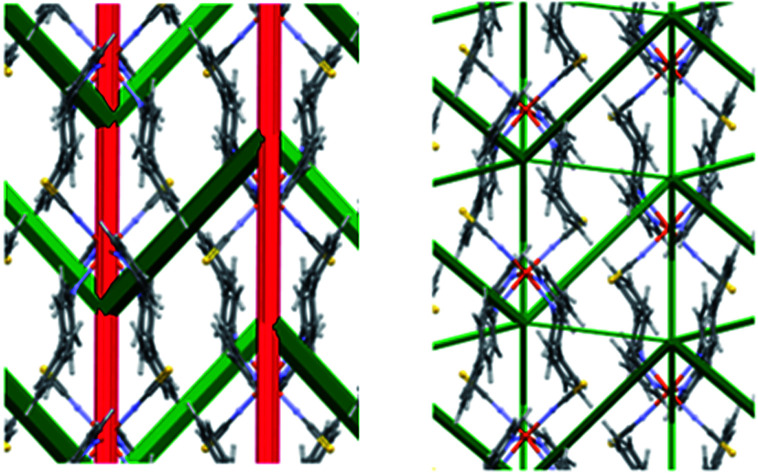

## 1. Introduction

In octahedral complexes in which the metal has a d^4^–d^7^ electronic configuration, the occupation of e_g_ and t_2g_ orbitals is directed by the nature of the ligand field. Weak field ligands result in a small energy difference between t_2g_ and e_g_ orbitals (Δ_O_), while strong field ligands lead to a larger Δ_O_. This leads to two possible electron distributions dependent on the size of Δ_O_ relative to the spin pairing energy (*E*_P_). Where *E*_p_ is greater than Δ_O_, d-electrons are distributed between the t_2g_ and e_g_ orbitals before pairing electrons to form a high spin configuration (HS). In contrast, where *E*_p_ is smaller than Δ_O_, electrons pair in the t_2g_ orbitals before occupying the higher energy e_g_ orbitals in a low spin (LS) state.^1–11^

In spin crossover (SCO) complexes, the values of Δ_O_ and *E*_p_ energies are similar, allowing a complex to exist as either HS or LS dependent on the amount of energy applied to a system in the form of temperature, pressure, or light. Such complexes can be reversibly switched between spin states, resulting in different magnetic, optical or structural properties. In thermally promoted spin crossover the low spin state is enthalpically favoured at low temperature, whereas the high spin state is entropically favoured at high temperature. SCO does not typically occur at a sharp, well-defined temperature, but instead occurs over a range of temperatures.

The transition temperature (*T*_1/2_), where the occupancies of molecules in the HS and LS states are equal, can be measured by several techniques including magnetic susceptibility, Mössbauer, Raman spectroscopies and X-ray structure determination.^2,11–16^ The abruptness of the transition represents the temperature range over which the crossover occurs and has been defined as the difference between the temperatures at which the HS state is 20 and 80% occupied (Δ*T*_60_, Fig. S1 in the ESI†).^17–24^ Abruptness is strongly related to the notion of cooperativity,^25^ the capability of one SCO centre to influence the spin state of a neighbouring one within the material. Abrupt spin crossover is also often associated with hysteretic behaviour, which may enable SCO complexes to be applied to information storage.^26–34^

The crossover from high to low spin may be accompanied by a change in the volume, in Fe(+2) complexes the result of depopulating the antibonding e_g_ orbitals. The volume change, which is usually anisotropic, generates strain, and a commonly adopted model of cooperativity links it to elastic interactions that propagate the volume change to the whole network,^21^ with strong interactions leading to a sharp spin crossover transition.^20–22,29^ A more recent approach correlates cooperativity with the change in the charge distribution along metal–ligand bonds, leading to an electrostatic contribution to cooperativity depending on the organisation of the metal–ligand dipole in a given crystal packing.^35^

Hence, cooperativity is a function of the crystal lattice and not of individual molecules,^22^ and substantially different SCO properties are possible for different polymorphs of the same complex (see below). The elastic and electrostatic models for cooperativity, which are not contradictory, highlight the need for the development of a quantitative understanding of the control of SCO transitions by intermolecular effects. This would represent a significant advance for the targeted design of new SCO materials.

A number of correlations based on the strength of specific contacts have been proposed for certain families of SCO complexes.^22,36–39^ Subtle interactions such as H⋯H contacts could play a crucial role in SCO phase transitions.^40^ In cases where a prominent interaction cannot be established, Hirshfeld surface analysis has been used to provide an overview of intermolecular bonding.^23,40,41^ The picture may be complicated because as key distances change across a SCO transition their influence on cooperativity changes also, so that the interactions which govern cooperativity in the HS to LS transition may be different from those in the LS to HS transition, leading to unsymmetrical hysteresis.

**Scheme 1:**
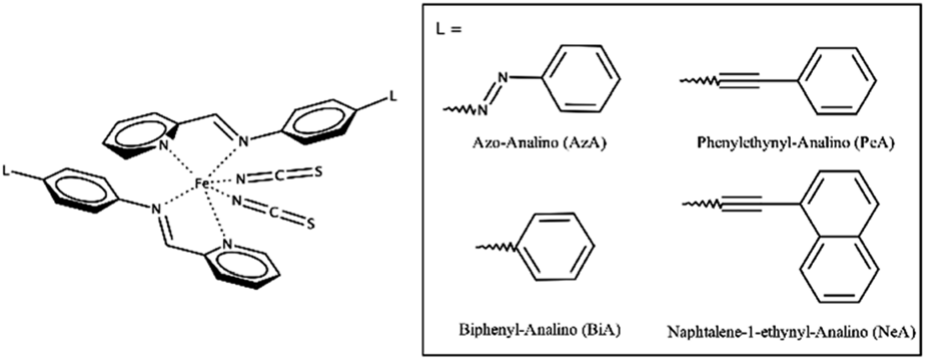
The general molecular structure of Fe(PM-L)_2_(NCS)_2_ complexes and the definition of PM-L ligands used in this study.

The cooperativity in Fe(+2) SCO complexes of general formula [FeL_2_(NCS)_2_] has been qualitatively shown to be a function of intermolecular π-stacking and other interactions.^42^ We will focus here on the relationship between intermolecular interactions and the SCO characteristics in a subclass of this family of general formula [Fe(PM-L)_2_(NCS)_2_] (Scheme 1). In this ‘PM-L’ family, Δ*T*_60_ can vary between 5 and 97 K. Differing behaviour is seen for two polymorphs of [Fe(PM-BiA)_2_(NCS)_2_] (PM-BiA = *N*-(2-pyridylmethylene)-4-aminobiphenyl), Δ*T*_60_ for one form being 5 K, but 81 K for the other, illustrating the importance of the crystal structure in determining SCO abruptness.^19,37,40^

**Fig. 1:**
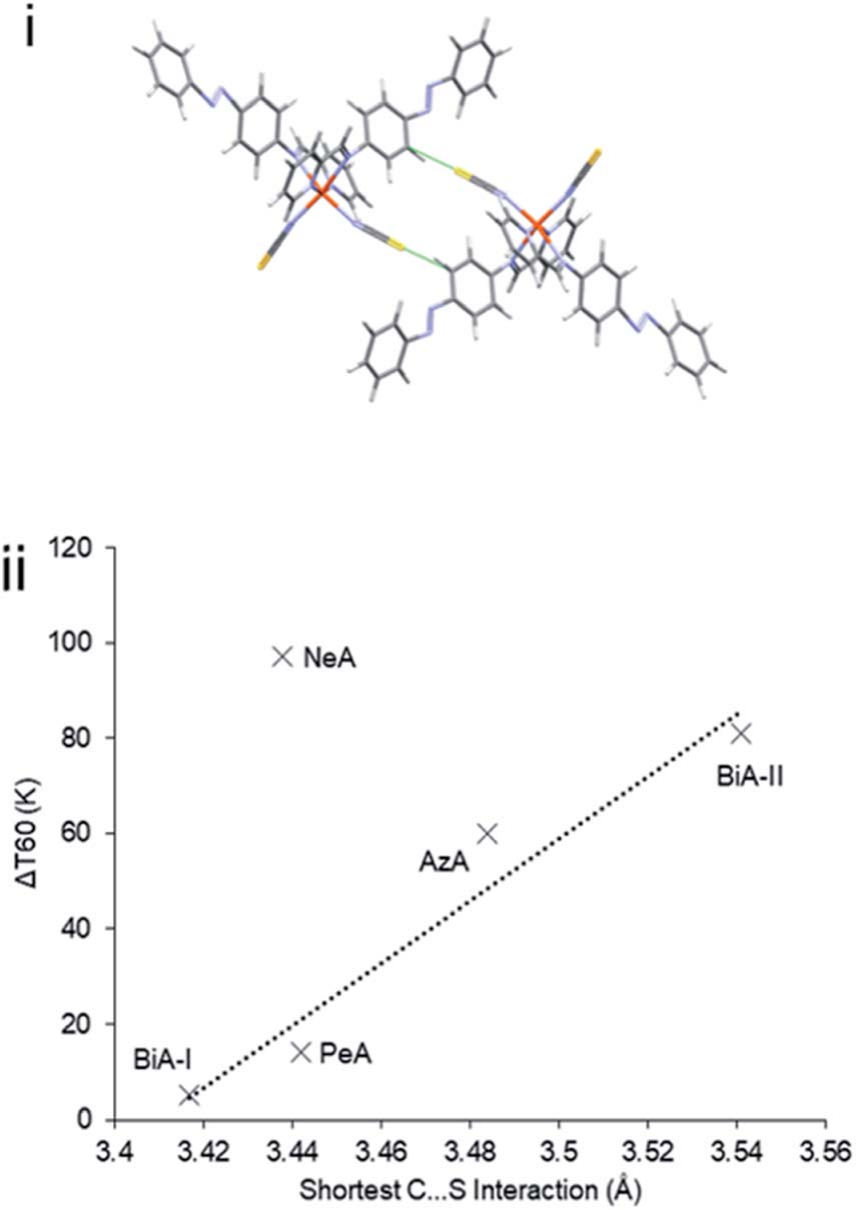
(i) S⋯H–C interactions in [Fe(PM-AzA)_2_(NCS)_2_]. (ii) Relationship between the S⋯C interaction distance and SCO transition abruptness (Δ*T*_60_) in the [Fe(PM-L)_2_(NCS)_2_] complexes of Scheme 1. The S⋯C distance is measured in the HS form in each case. BiA-I and BiA-II correspond to different polymorphs. The values plotted are available in Table S4.†

The abruptness of transitions in the PM-L family has been associated with a short S⋯H–C intermolecular contact in the crystal structures of the HS forms (Fig. 1i): where this contact is short the spin transition tends to be sharp (Fig. 1ii),^20^ suggesting that the strength of this interaction leads to increased cooperativity. While the trend is applicable to most members of the family, it does not apply to [Fe(PM-NeA)_2_(NCS)_2_] (NeA = naphthalene-1-ethynyl-anilino), which has short S⋯H–C contacts (S⋯C = 3.438 Å) but a very gradual transition (Δ*T*_60_ = 97 K).^43^

Packing energy calculations will be applied to the PM-L family with the aim of obtaining a more systematic and general overview of the energies of the intermolecular interactions which define the cooperativity pathways in these structures. The calculations have been performed with the PIXEL method,^44^ in which molecule–molecule (as opposed to atom–atom) energies are calculated semi-empirically using *ab initio* molecular electron densities. The calculations yield not only a total lattice energy, but also its breakdown into individual molecule–molecule contributions. Each total molecule–molecule energy (*E*_Tot_) is further decomposed into electrostatic (*E*_Elec_), polarisation (*E*_Pol_), dispersion (*E*_Disp_) and Pauli repulsion (*E*_Rep_) terms, providing insight not only on the strength, but also on the physical nature of the intermolecular interactions.

## 2. Methodology

### 2.1 Structural data

Four spin crossover complexes were investigated (Scheme 1). [Fe(PM-AzA)_2_(NCS)_2_] has a gradual SCO transition with Δ*T*_60_ = 60 K. Structures are available on the Cambridge Structural Database (CSD) for the HS form at 290 K (CSD Refcode: XECNAU35) and the LS form at 110 K (XECNAU07). The orthorhombic form-I of [Fe(PM-BiA)_2_(NCS)_2_] (HS, 290 K, RONPIT01; LS 140 K, RONPIT02) has an abrupt spin transition (Δ*T*_60_ = 5 K),^45^ while the monoclinic phase-II (HS, 290 K RONPIT04, LS 120 K RONPIT05) has a much more gradual transition (Δ*T*_60_ = 81 K).^19^ The temperature induced spin-transition of [Fe(PM-PeA)_2_(NCS)_2_] is accompanied by a phase transition upon heating/cooling from LS *Pccn* (140 K, NOWBIK) to HS *P*2_1_/*c* (290 K, NOWBIK01). The value of Δ*T*_60_ has been recently re-determined to be 14 K (the mean of 16 K on cooling and 12 K on warming). [Fe(PM-NeA)_2_(NCS)_2_] has the most gradual temperature-induced SCO transition found in the PM-L family (Δ*T*_60_ = 97 K). Structures for both HS (290 K, COMQUR) and LS (120 K, COMQUR01) forms are available.^43^ These data are summarised in Table S1 in the ESI.† Equivalent asymmetric units for all the HS and LS pairs of structures were obtained with the aid of the EQUIVSTRU utility on the Bilbao Crystallographic Server.^46–48^ The coordinates used for the calculations are available in electronic format in the ESI.† Interpretation of the crystal structures in terms of layer stacking was facilitated using the MechanicalProperties Python script described in ref. 49.

### 2.2 Pixel calculations

Lattice energies and intermolecular interaction energies were calculated using the semi-empirical computational technique PIXEL.^44,50,51^ PIXEL calculates energies by modelling each molecular component as a grid of small cubes (‘pixels’) of electron density. Interactions are calculated between a central reference molecule and other molecules within a cluster generated from the crystallographic space group symmetry. Intermolecular energies are calculated from the sum of electrostatic, polarisation, dispersion and (Pauli) repulsion terms accumulated from each pixel–pixel combination in a dimer. The sum of all cluster interaction energies gives the lattice energy. In this study the cluster radius was 20 Å, and the molecular electron densities were obtained in steps of 0.08 Å from GAUSSIAN-09 with the 6-31G** basis set at the B3LYP level of theory.^52^ The PIXEL calculations themselves were accomplished with the CLP-PIXEL suite within the MrPIXEL interface^53^ using a condensation level of 4 (*i.e.* the original pixels from GAUSSIAN were combined into 4 × 4 × 4 blocks of dimension 0.32 Å). The parameters applied to the transition metals were those derived by Maloney *et al.*^54^ C–H distances were reset to 1.089 Å to correct for the systematic shortening of bonds involving hydrogen in X-ray crystal structures. Note that the PIXEL method was originally devised for intermolecular energy calculations in crystal structures containing discrete molecules with no more than two complete entities in the crystallographic asymmetric unit. The implementation used for the present work reflects these limitations. However, the methods described in ref. 53 would enable calculations on more complex structures to be carried out, while those of ref. 55 would enable the method to be used for host–guest interactions in framework SCO materials.

### 2.3 Visualisation of results

Intermolecular interactions were visualised in Mercury^56^ using energy frameworks, originally devised by Spackman and co-workers.^57,58^ The width and colour of the struts drawn between molecules represent the intermolecular energy. In the figures below, green struts represent stabilising interactions (energy < 0), while red struts define destabilising interactions (energy > 0). The thickness of the strut represents the strength of the interaction. Further details are available in the ESI (Section S2†).

Comparisons can be made between crystal structures where interactions may be mapped from one structure to another, by calculating ‘difference frameworks’ where the strut sizes are related to the energy difference between the interactions in each structure. The program MrPIXEL, which is used to facilitate the Pixel calculations and generation of the frameworks, is available from http://www.crystal.chem.ed.ac.uk.

## 3. Results and discussion

### 3.1 Intermolecular interactions in [Fe(PM-L)_2_(NCS)_2_] crystal structures

The crystal structures in the [Fe(PM-L)_2_(NCS)_2_] family can be described in terms of the formation of layers in which the molecules are arranged so that a molecular axis drawn between the metal atom and the molecular centroid is parallel to the layer (ESI, Section S3†).^19,38,49,59^ The unit cell contains two offset layers in which the molecular axes point in opposite directions leading to an alternating stacking sequence. The closest layer spacing (−0.78 Å, Table S2†) occurs for the HS form of [Fe(PM-NeA)_2_(NCS)_2_], the negative sign indicating that there is some interpenetration between the layers (Fig. S3†).^49^ The largest (1.4 Å) occurs in [Fe(PM-BiA)_2_(NCS)_2_]-I LS (Fig. S4†).

**Fig. 2:**
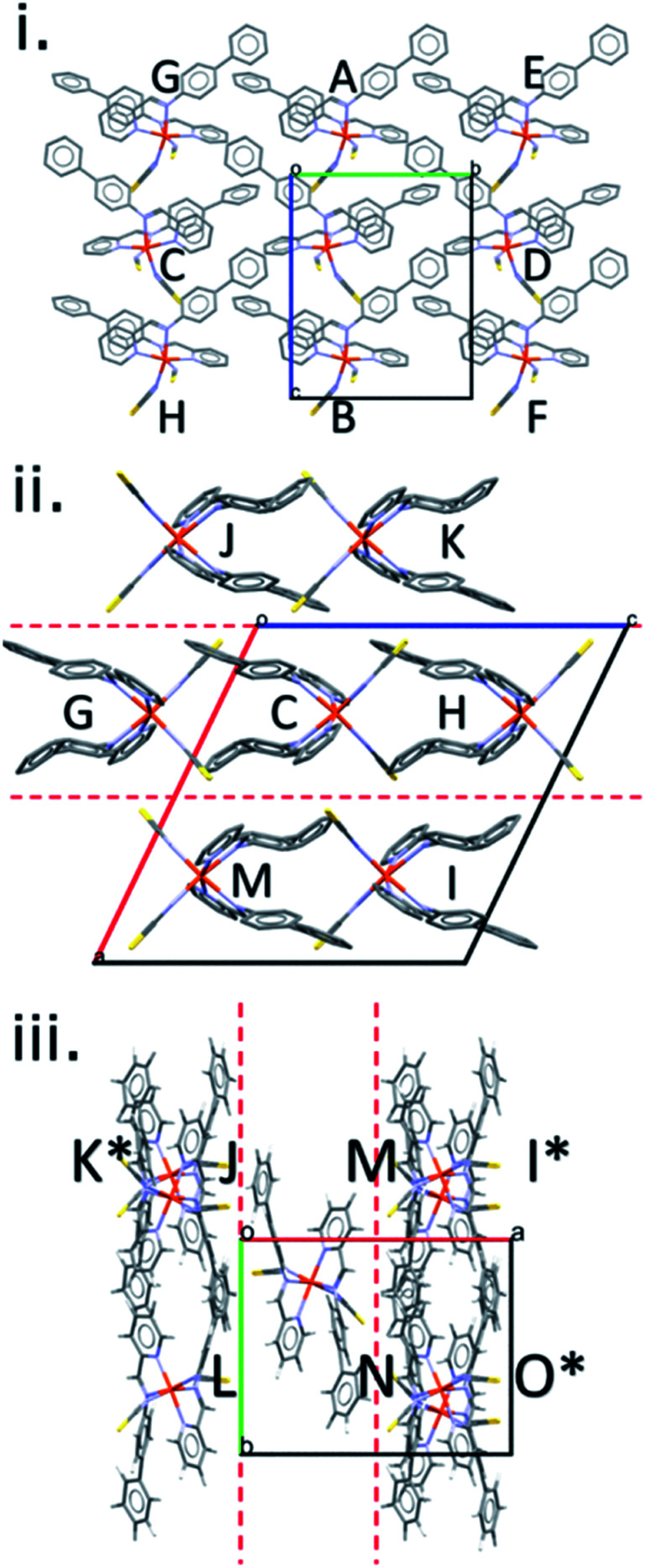
Stacking of layers in [Fe(PM-BiA)_2_(NCS)_2_] polymorph-II. Contacts to a central reference molecule are labelled A, B… layers (separated by red hashed lines), are viewed along the *a* (i), *b* (ii) and *c* (iii) axes, respectively. The reference molecule overlaps C in projection in (ii); where molecules are in background, labels are denoted with *.

The general features of packing and the pattern of intermolecular interactions in this family of spin crossover complexes can be illustrated using [Fe(PM-BiA)_2_(NCS)_2_] in polymorph-II of its high spin form (CSD Refcode RONPIT04, Fig. 2). Within a layer, each molecule makes energetically significant contacts with eight other molecules, labelled A–H in Fig. 2i and shown individually with their energy breakdowns in Table S3 in the ESI.† The shortest centroid–centroid distances (8.719 Å) occur along chains running parallel to the *c*-axis, interactions A and B in Fig. 2i. The molecule–molecule energy of these interactions is −97.9 kJ mol^−1^, and the shortest atom–atom contacts involve the thiocyanate sulfur atoms in one molecule and hydrogen atoms in the next (S1⋯H19, 2.86 Å). These interactions have large electrostatic and dispersion components. Adjacent chains in the same layer related by lattice translations along *b* interact more weakly (C/D, −20.9 kJ mol^−1^). The inter-chain contacts formed diagonally to molecules E/F and G/H in Fig. 2i have energies of −14.6 kJ mol^−1^ and −22.6 kJ mol^−1^, respectively. The largest contributing energy term in interactions C to H is dispersion.

There are seven interlayer interactions giving total molecular coordination numbers of 15. The contacts in [Fe(PM-BiA)_2_(NCS)_2_]-II (labelled I–O) vary in energy between −80.7 and −27.4 kJ mol^−1^. The strongest interactions, I and J, are formed across inversion centres and involve electrostatic thiocyanate⋯H and dispersion-dominated π⋯π interactions, respectively. In other contacts, the electrostatic term is largest where the closest atom–atom contacts involve the thiocyanate ligands; where the contact is between rings, dispersion dominates.

The shortest C⋯S contact (part of interaction denoted O), which has been implicated in controlling the SCO abruptness in previous work (see above), forms diagonally between molecules in adjacent layers (as shown in Fig. 2iii) for all structures, with thiocyanate group in one molecule pointing directly towards the phenyl H-atoms in the other.

It is thought that intermolecular contacts are the source of cooperativity in spin crossover transitions.^21,33^ However, the correlation between the energy of the intermolecular interaction mediated by this C⋯S contact and the SCO abruptness (Δ*T*_60_) was examined, but no simple trend could be identified (ESI, Section S4†). Likewise, consistent trends were absent for lattice energies, the lattice energy change over the course of the HS → LS transition and the layer spacing. Much more promising were trends based on energy frameworks.

### 3.2 Energy frameworks

Energy frameworks are a way of rapidly visualising intermolecular interactions in a crystal structure. The framework is constructed by linking pairs of molecules with struts, where the width of a strut is proportional to the intermolecular energy: thick struts correspond to strong interactions. Energy frameworks have been shown to be helpful, for example, in analysing the role of weak CH…halogen interactions on phase stability, host–guest interactions in clathrates, and the influence of the anisotropy of intermolecular interactions on the mechanical properties of organic solids.^57,58,60–66^

**Fig. 3:**
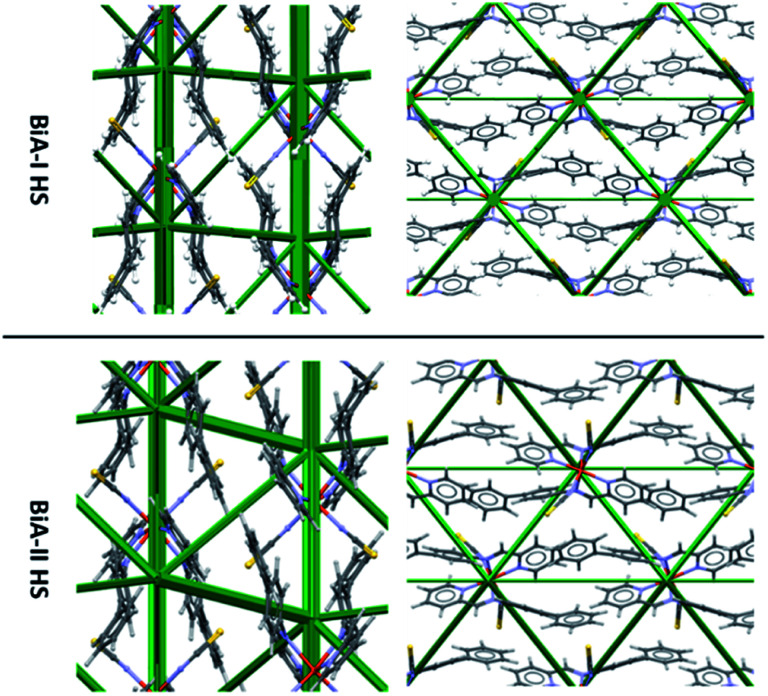
Comparison of HS energy frameworks for [Fe(PM-BiA)_2_(NCS)_2_] polymorphs I and II viewed along the *a* axis (left) and *c* axis (right). A comparison of the LS forms is shown in Fig. S6.†

However, the differences between the intermolecular interactions in the two polymorphs of PM-BiA-I are not at all obvious from a simple comparison of their energy frameworks (Fig. 3). As an alternative it is possible to produce an energy “difference” framework in which each strut represents a dimer interaction with a width proportional to its change in the total molecule–molecule energy *E*_Tot_ across the HS → LS transition, Δ*E* = *E*_Tot_(LS) − *E*_Tot_ (HS).

**Fig. 4:**
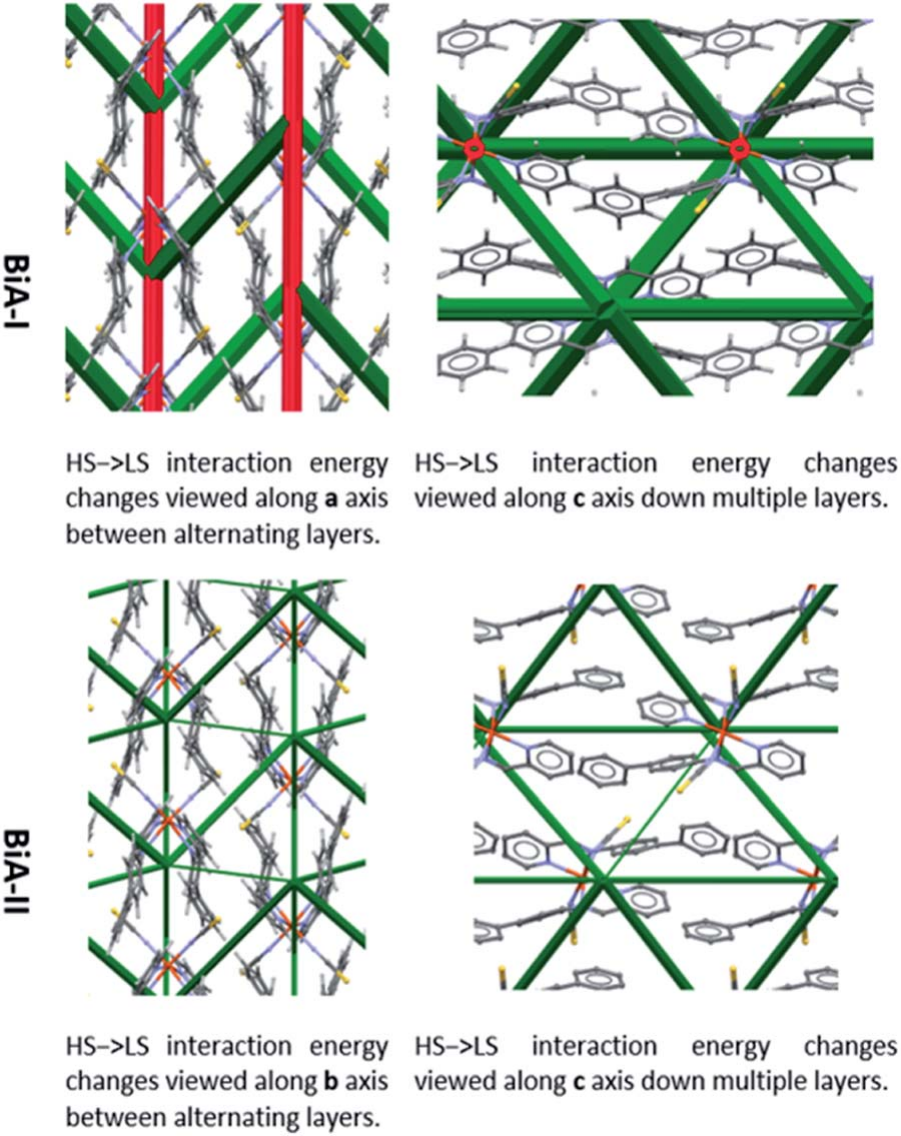
Energy difference frameworks for polymorphs of [Fe(PM-BiA)_2_(NCS)_2_]. For clarity, struts are only shown for the intermolecular first molecular coordination sphere (*i.e.* first nearest neighbours) where the interaction energy changes by more than 2.5 kJ mol^−1^. The same criteria apply to the other difference frameworks shown in this paper. Versions of these and other difference framework plots shown here which include the unit cell axes are available in the ESI, Fig. S7–S9.†

The difference frameworks for the two polymorphs of [Fe(PM-BiA)_2_(NCS)_2_] are shown in Fig. 4. Green struts correspond to interactions where the energy becomes more stabilising (*i.e.* Δ*E* < 0) during the HS to LS transition. Red struts show interactions which are destabilised (*i.e.* Δ*E* > 0). The magnitude of the energy change is shown by the thickness of the strut, as usual, but note that the scale factor relating energy and the width of the strut is different to that used in Fig. 3 (see ESI, Section S2† for details).

The difference framework of BiA-I, which has a very sharp transition, is characterised by thick stabilising (green) and destabilising (red) struts. In this structure interactions C, D, M and N are stabilised by over 10 kJ mol^−1^ over the course of the HS → LS transition, while interactions A and B, consisting of close S⋯H contacts, are destabilised (see Tables S6 and S7 in the ESI†). By contrast the difference framework of BiA-II, which has a broad SCO transition, has much thinner stabilising struts and no significant destabilising struts.

**Fig. 5:**
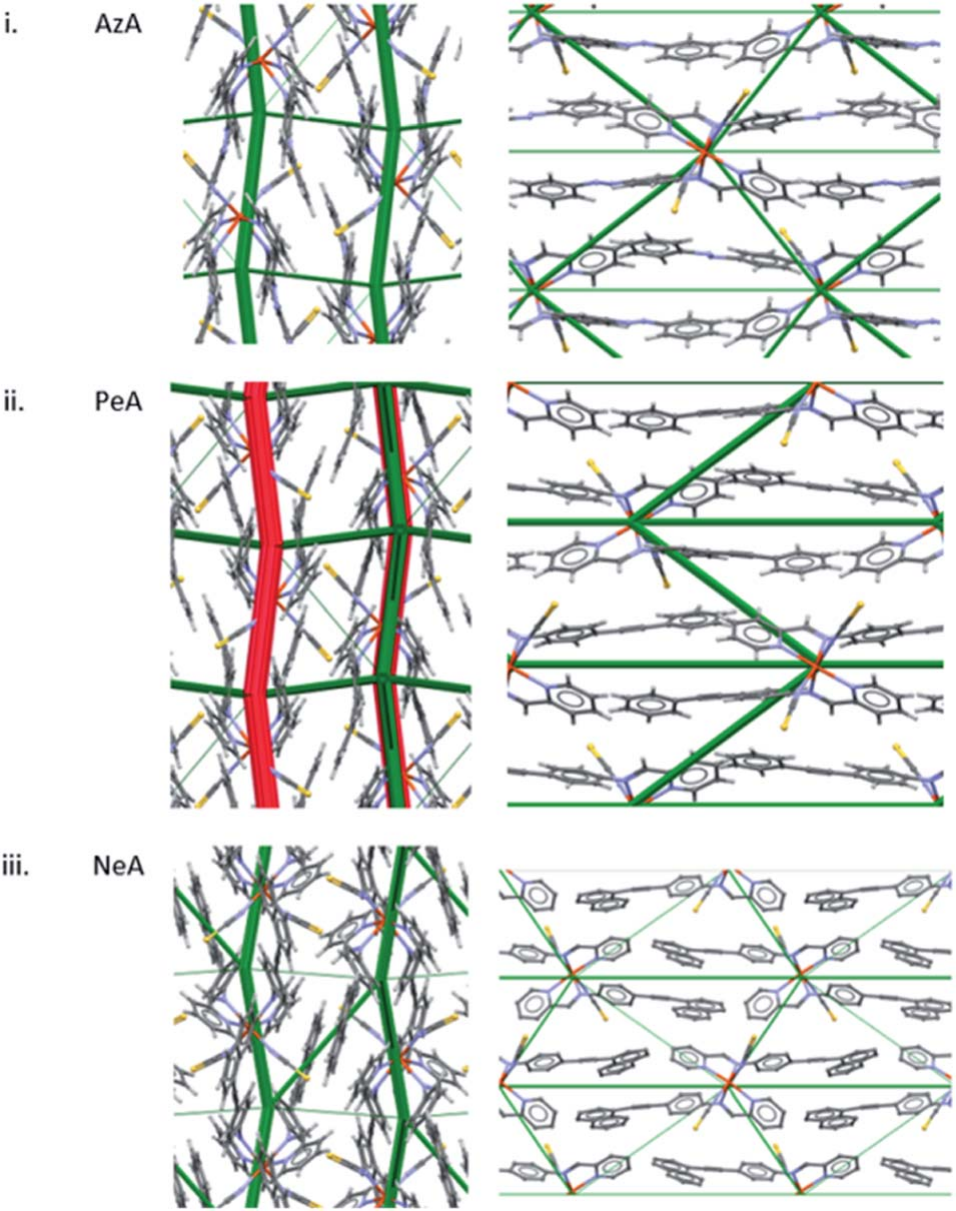
Energy difference framework for (i) [Fe(PM-AzA)_2_(NCS)_2_], (ii) [Fe(PM-PeA)_2_(NCS)_2_] and (iii) [Fe(PM-NeA)_2_(NCS)_2_]. The viewing directions are along the *a* (left) and *c* (right) axes.

A similar pattern emerges in other structures. Fig. 5i shows the energy difference framework for [Fe(PM-AzA)_2_(NCS)_2_] in which the generally thin green struts and the absence of red struts correctly suggests that it should, like [Fe(PM-BiA)_2_(NCS)_2_] form II, have a broad SCO transition. The energy difference framework for [Fe(PM-PeA)_2_(NCS)_2_] shows much larger energy changes between spin-states, with both stabilizing and destabilizing changes (Fig. 5ii), though these are less prominent than in the transition for [Fe(PM-BiA)_2_(NCS)_2_]-I. The transition is therefore expected to be more abrupt than for the AzA complex, but less so than for BiA-I. The [Fe(PM-NeA)_2_(NCS)_2_] complex undergoes the most gradual transition (97 K) in the PM-L family. As expected, the energy difference framework for this complex demonstrates much smaller energy changes between spin states, as shown in Fig. 5iii.

**Fig. 6:**
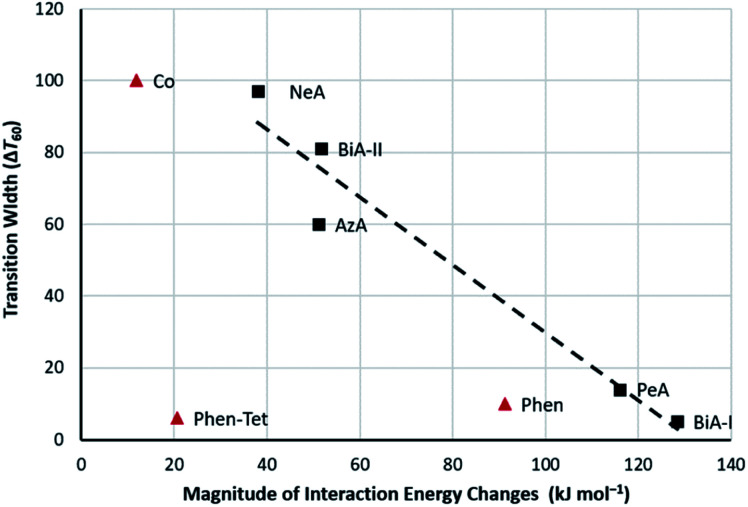
The transition abruptness (Δ*T*_60_ in K) plotted against the sum of the of absolute total interaction energy changes for [Fe(PM-L)_2_(NCS)_2_] SCO complexes (black squares) and three other SCO complexes (red trianges, see text). The equation of the fitted line is *y* = −0.9428*x* + 124.04.

The presence of prominent stabilising and destabilising energy changes is seen to be associated with a sharp SCO transition, while smaller changes and the absence of significant destabilising changes are associated with broad SCO transition. The relationship can be quantified in a plot of the sum of the magnitudes of all the interaction energy changes (∑|*E*_Tot_| = the sum of the widths of the struts shown in Fig. 4 and 5) against Δ*T*_60_, which is linear (Fig. 6) with a correlation coefficient (*r*) equal to −0.97. The correlation coefficient is negative because Δ*T*_60_ decreases as the energy changes increase. This linear trend is observed across the whole PM-L family, including the [Fe(PM-NeA)_2_(NCS)_2_] complex that did not follow the previous general trend based only on the C⋯S interaction,^43^ underlining the importance of considering the complete set of intermolecular interactions.

Linear trends are also seen for the most positive and most negative total interaction energy changes for each structure (Table S8†), corresponding the thickest red and green struts in the energy frameworks. Linear behaviour extends even to the magnitudes of the contributing energy terms themselves (also depicted in Fig. S10†), such as the sum of electrostatic energy magnitudes 
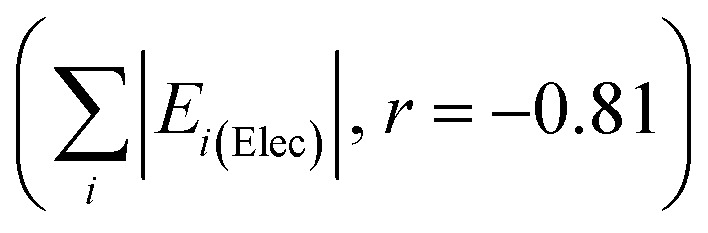
, the most positive total energy changes (Max *E*_Tot_, *r* = −0.95), and the most stabilizing total energy change (Min *E*_Tot_, *r* = 0.97). These correlations are much stronger than those found when signs of energy changes are taken into account (*r* = 0.45), which is consistent with the absence of a correlation between Δ*T*_60_ and the change in lattice energy (Fig. S5ii†).

The difference seen for the analysis of magnitudes and sums of energies may seem paradoxical, but abrupt SCO transitions are associated with the presence of thick struts in the energy frameworks which can correspond to large positive or negative energy changes. When signed energies are summed, large positive and negative energy changes can cancel each other out and their influence is lost in the total energy. The implication is that an abrupt transition is one in which the intermolecular interactions are able to accommodate the strain generated by the change in volume of the SCO complex in a flexible way, which may change individual terms substantially, but does not necessarily incur a large overall change in total energy. The role of the flexibility of the lattice in promoting cooperativity has been referred to previously,^22^ but the present results suggest that abruptness is associated with flexibility in specific interactions rather than in the crystal structure as a whole.

### 3.3 Application beyond the [Fe(PM-L)_2_(NCS)_2_] family

The results discussed so far have focussed on a single family of SCO complexes. Although the complexes are not strictly isostructural, the crystal packing and intermolecular interactions are consistent across the series, allowing direct comparison of interactions. The quantitative correlation shown in Fig. 6 would not be expected to extend generally, beyond the PM-L family, because different classes of SCO material will differ in polarity, molecular size and contacts, so that the scale of the intermolecular interaction energy changes will also be different.

**Fig. 7:**
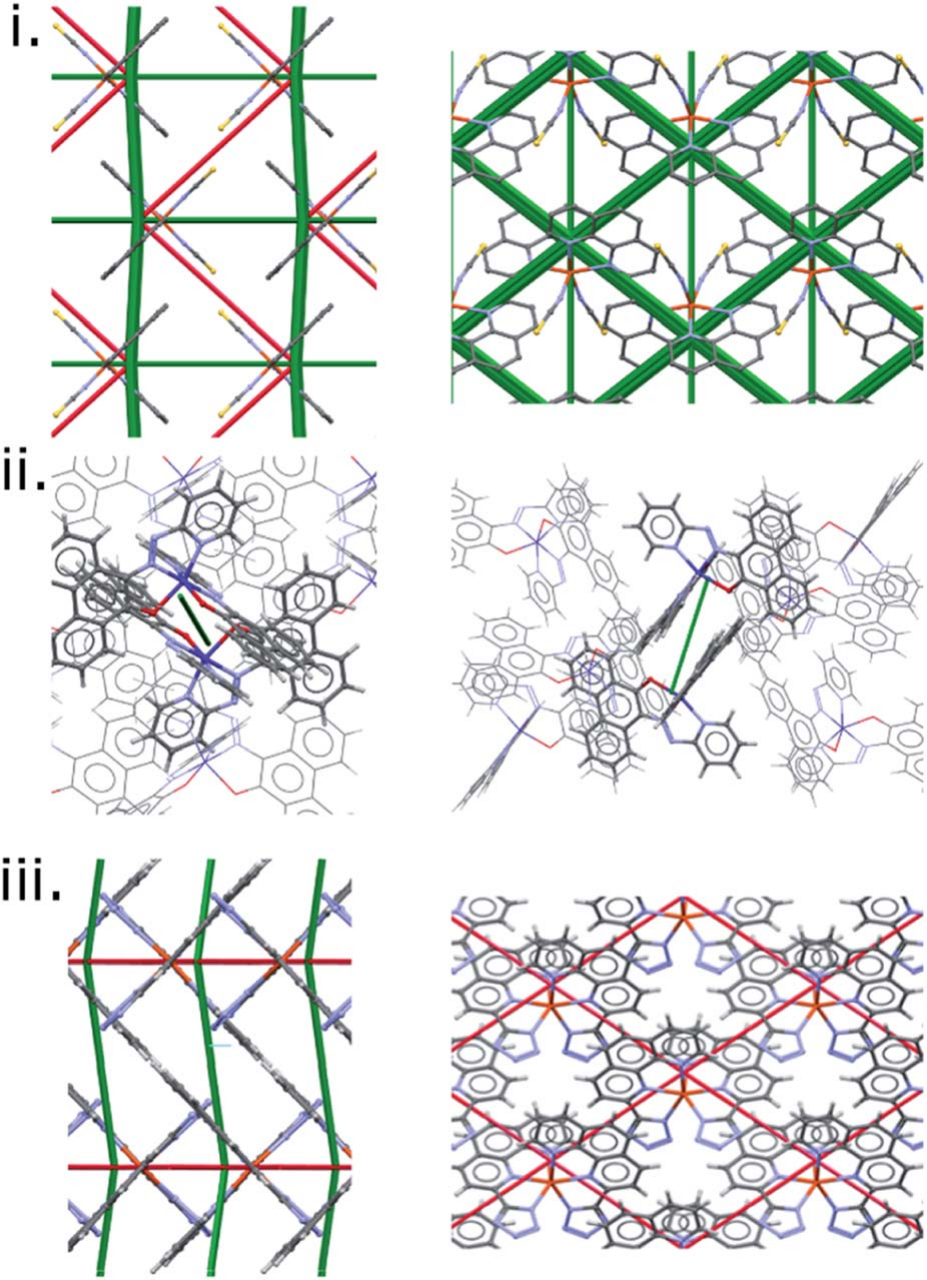
Energy difference frameworks for (i) [Fe(phen)_2_(NCS)_2_], (ii) bis(10-((pyridine-2-yl)diazenyl)phenanthrene-9-olato)-cobalt and (iii) [Fe(phen-Tetrazol)_2_].

The difference frameworks for three complexes from beyond the PM-L family are shown in Fig. 7, with energy data available in Table S9.† The complex [Fe(Phen)_2_(NCS)_2_] (‘Phen’, CSD Refcodes HS:KEKVIF, LS:KEKVIF01) is similar to the PM-L family in having thiocyanate ligands, but the phenanthroline ligands are smaller and better suited for graphitic stacking. The difference framework (Fig. 7i) consists of prominent green and red struts which are consistent with its abrupt SCO transition (Δ*T*_60_ = 10 K). In Fig. 6 the point for this complex lies close to, but not on, the PM-L correlation line.

In the cobalt complex PUYROS (‘Co’, HS: PUYROS01, LS: PUYROS) the metal binds to aromatic imines which are similar in some respects to PM-L ligands, but thiocyanates are absent. In common with most Co(2+) complexes, its SCO transition is very broad (Δ*T*_60_ is quoted as >100 K, but for the purposes of plotting we have used Δ*T*_60_ = 100 K). Accordingly, its difference framework (Fig. 7ii) is virtually featureless.

By contrast, [Fe(Tet-Phen)_2_(NCS)_2_] (‘Phen-Tet’, HS:QIDJET, LS:QIDJET01), which contains phenanthroline ligands substituted with anionic tetrazolyl groups, has a very abrupt transition (Δ*T*_60_ = 6 K), in line again with the prominent green and red struts found in its difference framework, but it does not fit the PM-L correlation at all (Fig. 6).

The value of Δ*T*_60_ for [Fe(Tet-Phen)_2_(NCS)_2_] is similar to that of [Fe(PM-BiA)_2_(NCS)_2_] form I, but the green and red struts of its framework are less prominent than those which characterise the plot for PM-BiA complex in Fig. 5i. Nevertheless, what the two plots do have in common is that they contain stabilising green and destabilising red struts of similar width. This observation is consistent with the trends seen in the PM-L family, in which prominent struts of both types are present in the more abrupt complexes.

**Fig. 8:**
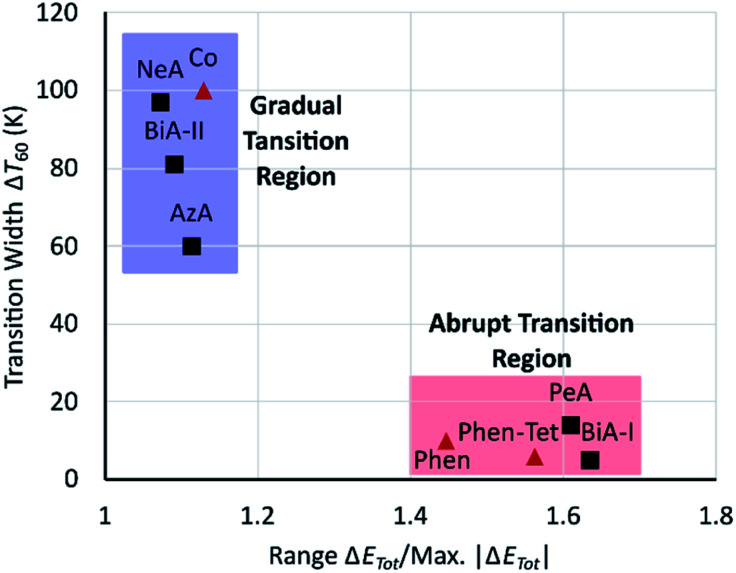
Transition width (Δ*T*_60_) against range Δ*E*_Tot_/max |Δ*E*_Tot_| for Fe(PM-L)_2_(NCS)_2_ SCO complexes (black squares) and other SCO complexes (red trianges). Range Δ*E*_Tot_ is defined as [most +ve Δ*E*_Tot_] − [most −ve Δ*E*_Tot_].

It seems that an abrupt transition requires both prominent stabilising and destabilising changes to be present, which is consistent with the suggestion, made above, that an abrupt transition is promoted by intermolecular interactions able to accommodate the strain generated by the change in volume of the SCO complex. Although the scale of these changes varies with the ligands, a plot of Δ*T*_60_ against the range of energy changes (range Δ*E*_Tot_) normalised by dividing by the magnitude of the maximum interaction energy change (*i.e.* the width of the thickest strut) (Fig. 8) shows that fast and slow transitioning complexes cluster in two different regions. The normalisation addresses the non-fitting compounds in Fig. 6, emphasising the importance of global evaluation of intermolecular interactions, and suggesting that the approach described here can be applied to more generally SCO compounds.

## 4. Conclusions

The aim of this work has been to explore the relationship between the abruptness of spin-crossover transitions and intermolecular interaction energies. Only crystalline solids have been considered. The study has concentrated on the PM-L family of Fe(2+) complexes. The packing is consistent within this series of crystal structures, enabling direct comparisons to be made between different members of the family. A full set of structural and magnetic data is also available for both HS and LS forms of all complexes. The crystal structures can also be described with one molecule in the asymmetric unit (lowering symmetry where necessary), making them suitable for lattice energy calculations using PIXEL. Limitations in the current implementation of the PIXEL method have been described in the Experimental section.

No correlation was found between abruptness and lattice energy, the intermolecular energy involving shortest HS C⋯S contact, or the changes in layer stacking which occur across an SCO transition. A more consistent trend emerges by considering the changes in individual intermolecular interaction energies, and there is a linear variation in Δ*T*_60_ with the sum of the magnitudes of the interaction energy changes within the first molecular coordination sphere (Fig. 6). These changes can be visualised in energy difference frameworks, which could also be considered as illustrating the cooperativity pathways of an SCO transition.

Abrupt spin crossover transitions require some large stabilising and destabilising changes in intermolecular energies. It does not seem to matter which interactions are involved: the largest positive and negative energy changes depend on the structure, and, despite the packing similarities across the PM-L family, there is no consistency in which interaction shows the largest energy change.

The quantitative trend established for the PM-L family does not directly extend to other classes of SCO complexes, and we have suggested that this is because the scale of the energy changes which occur are strongly dependent on the ligands present. Instead, it is necessary to assess the significance of individual contact energy changes in the context of the overall magnitude of the changes taking place. A clear correlation was found, independent of the chemical family, when normalized interaction energy changes were used against the abruptness of the transition with sharp and broad transitioning complexes clustering together in different regions of a plot of Δ*T*_60_ against range Δ*E*_Tot_/max |Δ*E*_Tot_| (Fig. 8).

Our analysis is based on a comparison of the two end-member spin states, represented by the high and low spin crystal structures. This approximation would fail, for example, in the case of an SCO transition displaying an unsymmetrical hysteresis, in explaining the differences which occur between single crystals and polycrystalline forms of SCO materials where local and surface effects are influential, or in explaining the effect of thermal history. Modelling intramolecular bond energy changes to allow the method to be applied to propagation of SCO in frameworks would require changing the methodology used for the calculations to one based on quantum mechanics.

Nevertheless, the results suggest that the abruptness of an SCO transition is related to the accommodation of strain which is generated as the volumes of individual molecules change with spin state. Large values of ∑|*E*_Tot_| (thick struts) indicate that strain can be accommodated in specific interactions instead of needing to be propagated through the entire structure, destabilising changes being compensated for by stabilising changes. For broad transitions, the small energy changes and lack of compensating energy changes appear to cause a slower propagation of spin transitions through the system.

We have used energy frameworks calculated from crystal structure data to obtain 3D maps which yields a broad overview of the changes in the strengths and physical characteristics of intermolecular interactions that occur over an SCO transition. The approach shifts the focus from specific inter-atomic contact lengths and angles to interactions involving whole molecules, providing a new and generally applicable perspective in the understanding of the relationship between structure and properties in SCO materials.

Although SCO materials have been extensively investigated for over 30 years, there are still many fundamental questions to be addressed. Amongst these is a precise understanding of the concept of cooperativity.^21,35^ Energy frameworks have the potential to reveal the pathways and mechanisms of cooperativity, a breakthrough which would allow, in a further step, the rational design of SCO materials with truly technologically relevant features.

## Conflicts of interest

There are no conflicts to declare.

## Supplementary Material

SC-012-D0SC05819J-s001

SC-012-D0SC05819J-s002

SC-012-D0SC05819J-s003

SC-012-D0SC05819J-s004

SC-012-D0SC05819J-s005

SC-012-D0SC05819J-s006

SC-012-D0SC05819J-s007

SC-012-D0SC05819J-s008

SC-012-D0SC05819J-s009

SC-012-D0SC05819J-s010

SC-012-D0SC05819J-s011

SC-012-D0SC05819J-s012

SC-012-D0SC05819J-s013

SC-012-D0SC05819J-s014

SC-012-D0SC05819J-s015

SC-012-D0SC05819J-s016

SC-012-D0SC05819J-s017

SC-012-D0SC05819J-s018

SC-012-D0SC05819J-s019

SC-012-D0SC05819J-s020

SC-012-D0SC05819J-s021

SC-012-D0SC05819J-s022

SC-012-D0SC05819J-s023

SC-012-D0SC05819J-s024

SC-012-D0SC05819J-s025

SC-012-D0SC05819J-s026

SC-012-D0SC05819J-s027

SC-012-D0SC05819J-s028

SC-012-D0SC05819J-s029

SC-012-D0SC05819J-s030

SC-012-D0SC05819J-s031

SC-012-D0SC05819J-s032

SC-012-D0SC05819J-s033
