# Supplementary material for: Mapping the cooperativity pathways in spin crossover complexes
Source: Chem Sci. 2020 Nov 16;12(3):1007–15. doi: 10.1039/d0sc05819j (PMC8179037; doi:10.1039/d0sc05819j)
Supplement: SC-012-D0SC05819J-s033 [file SC-012-D0SC05819J-s033.pdf]

Electronic Supplementary Material (ESI) for Chemical Science.
This journal is © The Royal Society of Chemistry 2020

SUPPLEMENTARY INFORMATION

Mapping the Cooperativity Pathways in Spin Crossover Complexes

Matthew G. Reeves, Elodie Tailleur, Peter A. Wood,* Mathieu Marchivie,* Guillaume Chastanet, Philippe Guionneau,* and Simon Parsons*

Contents

Section S1: Parameter definitions and data selection. (Page 2-3)

Section S2: Supplementary details on energy framework generation. (Page 4)

Section S3: Further details on packing in the crystal structures of $[Fe(PM\text{-}L)_2(NCS)_2]$ complexes. (Page 5-8)

Section S4: The Relationship of SCO behaviour to structural parameters and PIXEL energies. (Page 9-11)

Section S5: Further energy framework plots. (Page 12-15)

Section S6: Data used for generation of energy difference frameworks. (Page 16-35)

Section S1: Parameter definitions and data selection

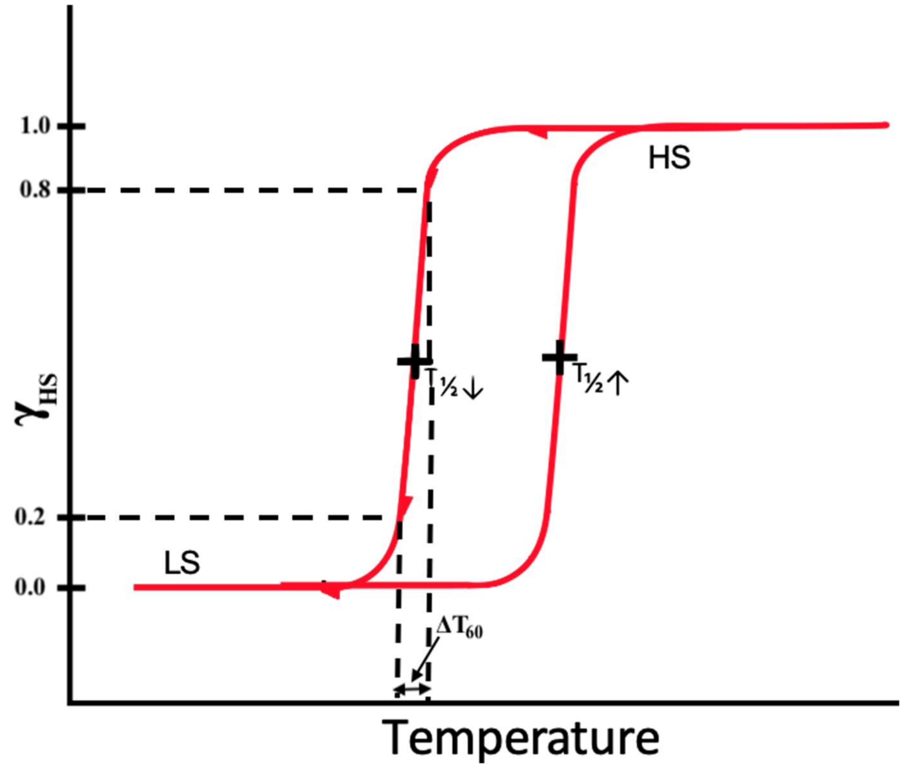

Figure S1: Hysteresis loop for temperature−induced SCO behaviour. Arrows show direction of spin transition. In case of hysteresis, two values of $T_{1/2}$ exist, corresponding to the ascending (↑) and descending (↓) branches. The abruptness of the transition represents the temperature difference between the 20 and 80% HS state rate and is denoted ΔT_{60}. Where a hysteresis is present, two values of ΔT_{60} exist according to the sharpness of the transition during the cooling and the warming modes. Here only the HS−>LS transition abruptness ΔT_{60}↓ is shown.

Table S1: Selected data for complexes studied. The refcode refers to the entry in the Cambridge Structural Database. *T* = temperature of the crystal structure determination; ΔT_{60} is defined in Fig. S1. Scheme 1 from the main text is reproduced below for convenience.

**Complex**	**Spin state**	**Refcode**	**T/K**	**ΔT_{60}/K**	**Space Group**
**BiA-I**	HS	RONPIT01	290	5	*Pccn*
	LS	RONPIT02	140		*Pccn*
**PeA**	HS	NOWBIK01	290	14	$P2_1/c$
	LS	NOWBIK	140		*Pccn*
**AzA**	HS	XECNAU35	290	60	$P2_1/c$
	LS	XECNAU07	110		$P2_1/c$
**BiA_II**	HS	RONPIT04	290	81	$P2_1/c$
	LS	RONPIT05	120		$P2_1/c$
**NeA**	HS	COMQUR	290	97	$P2_1/c$
	LS	COMCUR01	120		$P2_1/c$

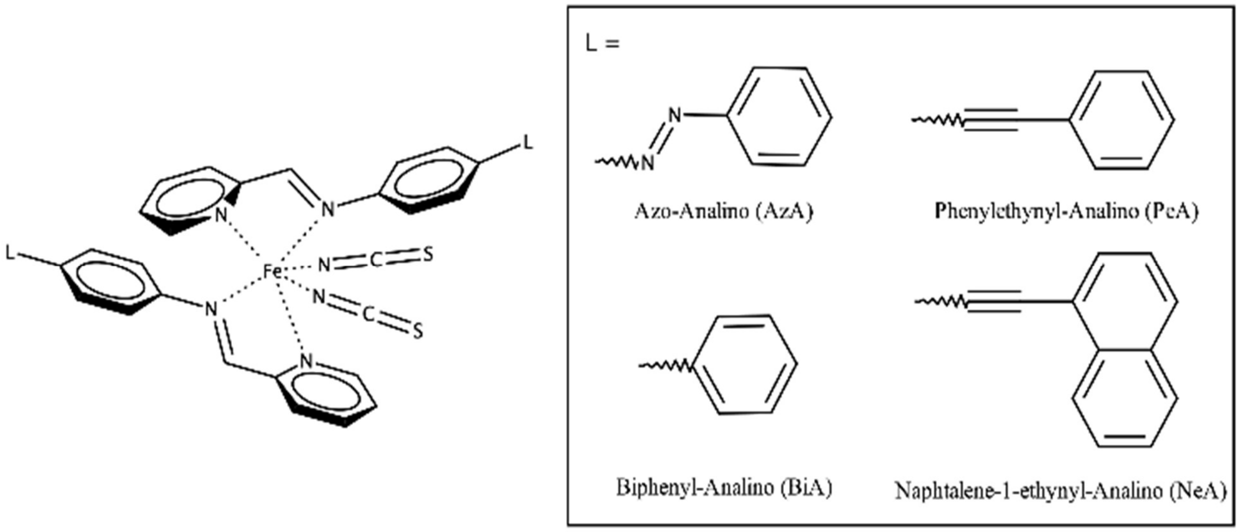

Scheme 1: $Fe(PM\text{-}L)_2(NCS)_2$ Complex. PM-L Ligands for studied structures.

Section S2: Supplementary details on energy framework generation

The frameworks were generated using the CSD Python API (application programming interface). A vector M_a-M_b defined between the molecular centroids of each interaction identified in a PIXEL calculation (Fig. S2). An atom is placed at the mid-point of the vector, M_p; to enable colour-coding of the framework struts, which are drawn as coordination polyhedra, radium was used for stabilising contacts and meitnerium destabilising contacts. Six deuterium atoms are placed orthogonal to the vector M_a-M_b and at a distance E/n from both M_a and M_b. E is the energy of the interaction in kJ mol^{-1} and n scales the diameter of the struts of the framework. Direct visual comparisons between frameworks can be made provided the same value of n is used; in this work n was chosen to be 200 for energy frameworks and 25 for energy difference frameworks. The struts are then constructed by representing the atom at M_p as a hexagonal prism with the vertices at the deuterium atoms. The updated structure is saved as a .MOL2 format file, which can then be visualised in Mercury. As has been described by Spackman, frameworks may be generated for each separate component of the interaction energy (electrostatic, polarisation, dispersion, repulsion) or as the total.

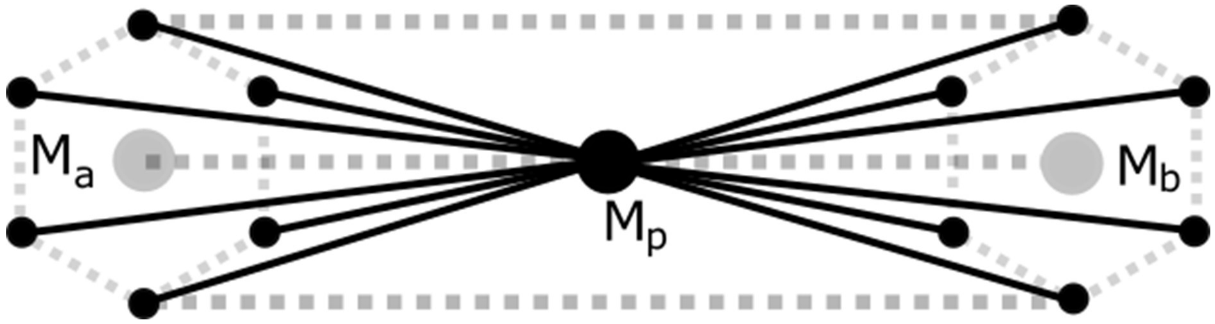

Figure S2: Example of strut construction showing new atoms placed between the centroids of two molecules at Ma and Mb and bonds between newly placed atoms.

Section S3: Further details on packing in the crystal structures of [Fe(PM-L)$_2$(NCS)$_2$] complexes

Shown here are high-spin Fe(PM-NeA)$_2$(NCS)$_2$ and Fe(PM-BiA)$_2$(NCS)$_2$ polymorph-II which correspond to the smallest and largest layer separation respectively. Quantitative values of layer spacing are displayed in Table S1.

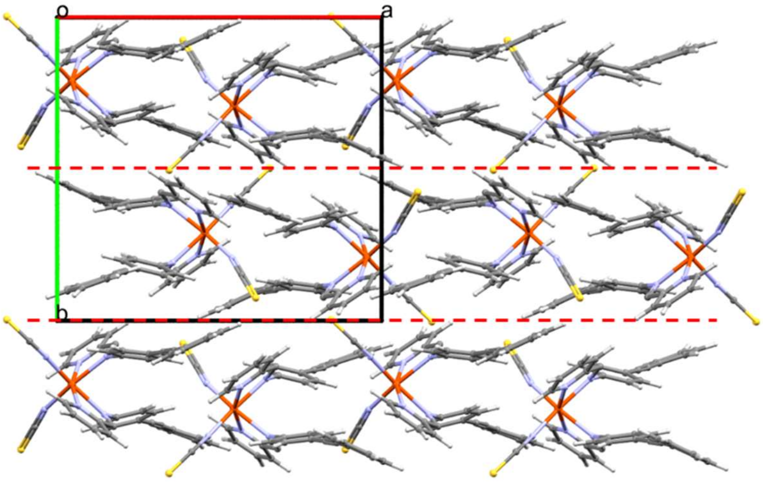

Figure S3: View of the layer structure of HS Fe(PM-NeA)$_2$(NCS)$_2$ along **c**.

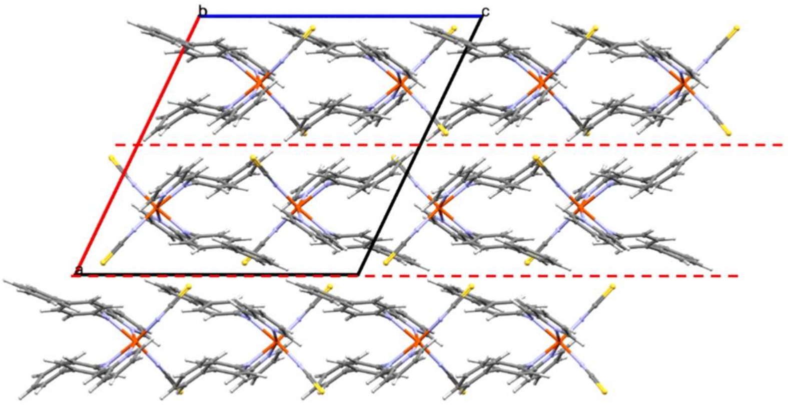

Figure S4: View of the layer structure of HS Fe(PM-BiA)$_2$(NCS)$_2$ polymorph-II along **b**.

Table S2: Layers in SCO structures. Values determined using the topological analysis scripts developed by Bryant et. al.[1]

Structure	Refcode	Slip Plane [h,k,l]	Layer Spacing (Å)
**Fe(PM-AzA)$_2$(NCS)$_2$ HS**	XECNAU35	[0,1,0]	0.10
**Fe(PM-AzA)$_2$ (NCS)$_2$ LS**	XECNAU07	[0,1,0]	0.02
**Fe(PM-BiA)$_2$ (NCS)$_2$–I HS**	RONPIT01	[0,2,0]	0.12
**Fe(PM-BiA)$_2$ (NCS)$_2$–I LS**	RONPIT02	[0,2,0]	1.40
**Fe(PM-BiA)$_2$ (NCS)$_2$–II HS**	RONPIT04	[1,0,0]	0.92
**Fe(PM-BiA)$_2$ (NCS)$_2$–II LS**	RONPIT05	[1,0,0]	1.06
**Fe(PM-PeA)$_2$ (NCS)$_2$ HS**	NOWBIK01	[0,1,0]	−0.10
**Fe(PM-PeA)$_2$ (NCS)$_2$ LS**	NOWBIK	[2,0,0]	0.50
**Fe(PM-NeA)$_2$(NCS)$_2$ HS**	COMQUR	[0,1,0]	−0.78
**Fe(PM-(NeA)$_2$(NCS)$_2$ LS**	COMQUR01	[0,1,0]	−0.74

Table S3: First co–ordination sphere interactions (in kJ mol^{-1}) for HS $Fe(PM\text{-}BiA)_2(NCS)_2$ polymorph-II

Interaction	Centroid-Centroid Distance	Symmetry	E_{Elec}	E_{Pol}	E_{Disp}	E_{Rep}	E_{Tot}	Description	Figure
**Intra–Layer**									
**A/B**	8.719	x,−y+½,z−½<br>x,−y+½,z+½	−57.4	−28.8	−80.4	68.6	−97.9	Interaction along chain in a single layer with overlap of NCS and PM−X ligands	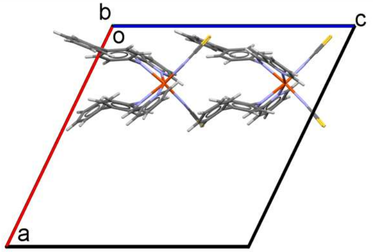
**C/D**	12.602	x,y−1,z<br>x,y+1,z	9.6	−5.9	−44.2	19.6	−20.9	Adjacent complexes in same layer with same orientation of ligands and molecular axis.	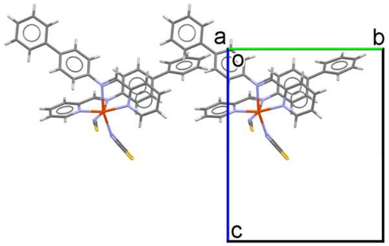
**E/F**	15.996	x,3/2−y, z−1/2<br>x,3/2−y, z+1/2	−6.5	−5.0	−11.5	8.4	−14.6	Diagonal complexes in same layer with same molecular axis and opposite ligand orientations.	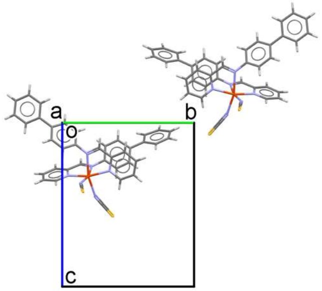

**G/H**	14.621	x,−y−1/2 ,z+1/2 x,−y−1/2, z−1/2	−9.1	−7.2	−18.8	12.6	−22.6	Diagonal complexes in same layer with same molecular axis and opposite ligand orientations. Typically, shorter than E/F contacts.	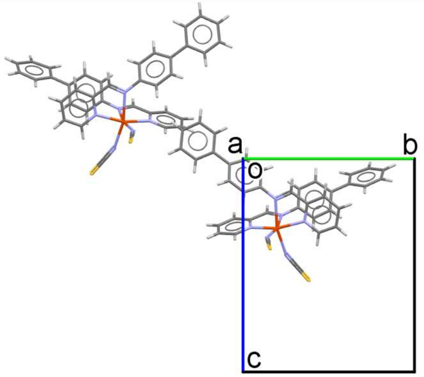
**Inter−Layer**									
**I**	12.318	1−x, −y,1−z	−39.0	−8.0	−12.6	7.9	−51.7	Diagonal contact between layers with thiocyanate ligands pointing away from the contact vector. 2 short NCS…PM contacts (4.035 Å).	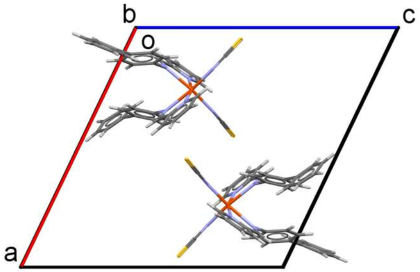
**J**	9.833	−x, −y, −z	−27.7	−14.7	−77.7	39.4	−80.7	Adjacent interlayer contact with large overlap of PM−R group aromatic rings, resulting in a strong dispersion term.	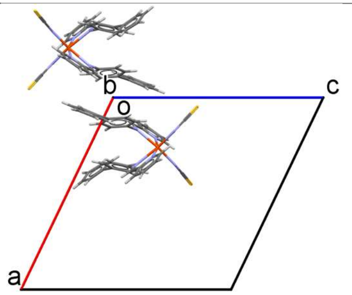

**K/L**	12.090	−x, y+1/2, 1/2−z<br>−x, y−1/2, 1/2−z	−34.2	−12	−21.3	20.1	−47.5	Diagonal contact between layers with short NCS...R group contacts.	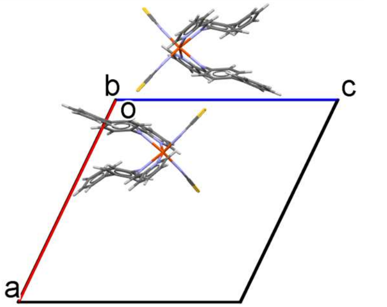
**M/N**	10.171	1−x, y−1/2, 1/2−z<br>1−x, y+1/2, 1/2−z	−17.2	−11.1	−44.6	28.2	−44.7	Adjacent interlayer contact similar to Interaction J but with poorer aromatic ring stacking.	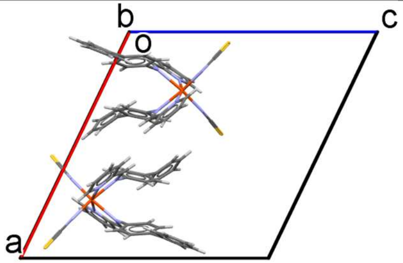
**O**	13.145	1−x, 1−y, 1−z	−14.8	−12.6	−23.5	23.5	−27.4	Diagonal contact across layers with NCS groups orientated along the contact vector. Shortest C...S interaction.	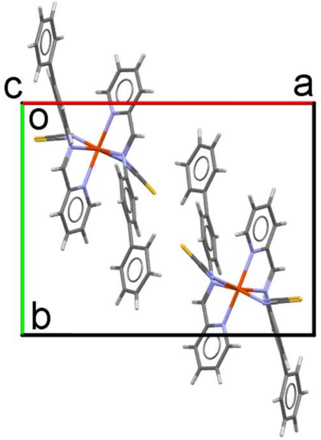

Section S4: The relationship of SCO behaviour to structural parameters and PIXEL energies

In the following sections we examine the correlations of shortest C…S distance, lattice energy and layer stacking with the SCO transition abruptness in the $Fe(PM\text{-}L)_2(NCS)_2$ family of complexes.

The shortest C…S Interaction

The suggestion that spin transition abruptness is related to the strength of the intermolecular interactions mediated by the short C…S contacts may be analysed in terms of interaction energies using the PIXEL results for the interaction denoted O (see Fig. 2iii in the main text) and its analogues in other structures (see also Table S4). While there are substantial differences in intermolecular interaction energies in these systems, no simple trend can be identified between the transition abruptness (ΔT_{60}) and either the total dimer energy in the high or low spin forms or the change in dimer energy between spin states (Fig. S5i). We suggest that SCO behaviour cannot be fully described or predicted from the energy of the C…S interaction, and we therefore investigated whether parameters such as lattice energy or inter-layer spacing, which are features of the whole crystal structure, might be more effective.

Lattice Energies

The lattice energies calculated using PIXEL are available for each structure and are listed in Table S5. The lattice energy change between spin-states at 110 and 290 K is in the order of –20 to –50 kJ mol^{-1} for all complexes, with the two polymorphs of $Fe(PM\text{-}BiA)_2(NCS)_2$ having very similar lattice energies for both spin states (E_L(HS) and E_L(LS), respectively). This may explain why both polymorphs are observed under ambient conditions, though the energies are too similar to state with confidence which is the more stable form. From these results there is no clear correlation between transition width and the lattice energies of either the HS or LS structures. Neither is there a correlation with change in lattice energy between spin-states for the HS → LS transition, defined as $\Delta E_L = E_L(LS) - E_L(HS)$ (Fig. S5ii).

Layer Spacing

Previous studies find no clear trend relating to the isotropic cell contraction (ΔV_{SC}) and the SCO characteristics, but do point towards the anisotropy of the cell contraction as a parameter relating to the abruptness of transition.[2] The change in layer spacing is a component of the anisotropic cell volume contraction and thus provides information on the nature of crystal packing changes in relation to SCO behaviour for $Fe(PM\text{-}L)_2(NCS)_2$ with this layered packing. Although there is no apparent trend between the level of interpenetration of the layers described above and the abruptness of transition, the change in layer separation between spin-states does suggest that large negative changes in layer separation correlate with sharper SCO (Fig. S5iii), though this correlation does not extend to the broader transitions.

Table S4: Shortest C…S contact distances and energies in the HS forms

.

PM–X Group	ΔT_{60} (K)	Shortest HS C…S Distance (Å)	Shortest LS C…S Distance (Å)	HS Symmetry Operation	HS Interaction Energy (kJ mol^{-1})	LS Interaction Energy (kJ mol^{-1})	ΔE_{LS-HS} (kJ mol^{-1})
**BiA–I**	5	3.417	3.436	2−x,−y,−z	−37.8	−40.6	−2.8
**PeA**	14	3.447	3.585	2−x,1−y,z	−21.0	−42.0	−21.0
**AzA**	60	3.484	3.411	1−x,−y,−z	−20.0	−20.1	−0.1
**BiA–II**	81	3.541	3.450	1−x,1−y,1−z	−27.4	−31.8	−4.4
**NeA**	97	3.438	3.445	−x,1−y,1−z	−31.6	−33.7	−2.1

Table S5: Overall PIXEL lattice energies (in kJ mol^{-1}) for the $Fe(PM\text{-}L)_2(NCS)_2$ family of SCO complexes.

PM–X Group	ΔT_{60} (K)	PIXEL Lattice Energies (40 Å Cut–off)		
		HS $E_{Lattice}$	LS $E_{Lattice}$	$\Delta E_{Lattice}$
**BiA–I**	5	−300.5	−327.8	−27.3
**PeA**	14	−300.4	−351.9	−51.5
**AzA**	60	−319.3	−342.6	−23.3
**BiA–II**	81	−297.9	−327.8	−29.9
**NeA**	97	−352.8	−372.7	−19.9

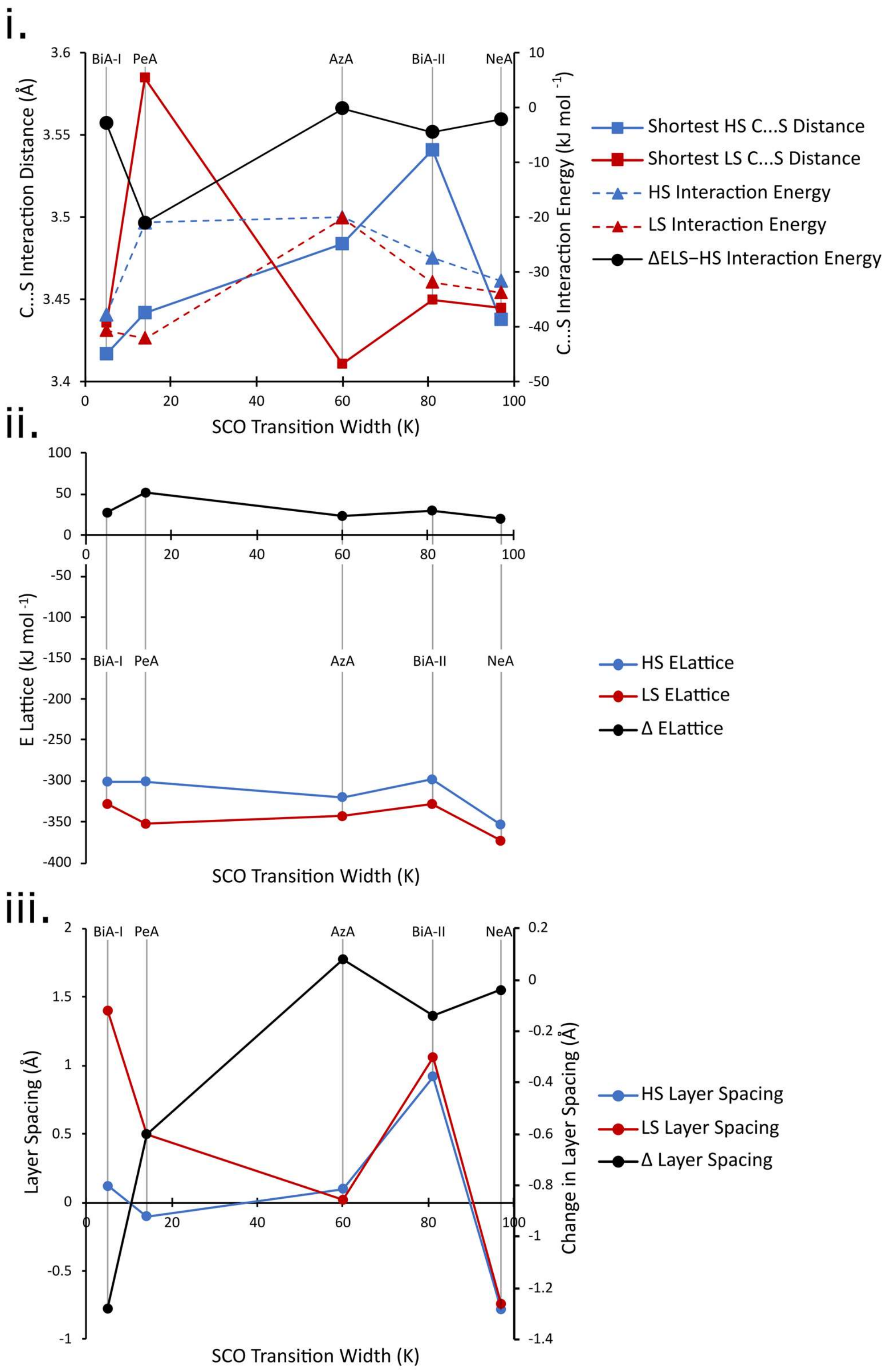

Figure S5: **i.** SCO transition width against the shortest C..S interaction distances and PIXEL interaction energies. Data for both HS and LS forms are plotted. **ii.** SCO transition width against HS/LS PIXEL lattice energies. **iii.** SCO transition width against HS and LS layer spacing

Section S5: Further energy framework plots.

BiA-I LS

BiA-II LS

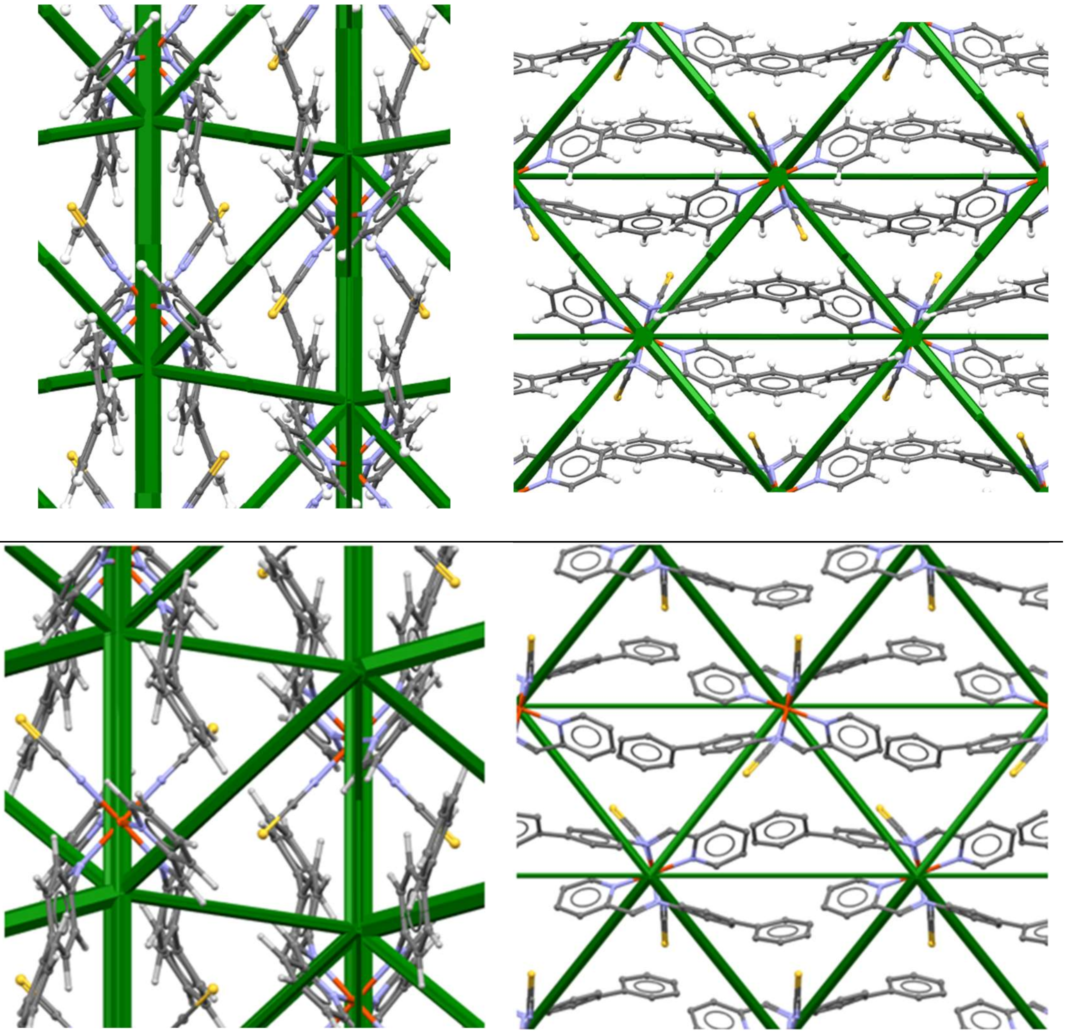

Figure S6: Comparison of LS energy frameworks for $Fe(PM\text{-}BiA)_2(NCS)_2$ polymorphs I and II viewed along *a* axis (left) and *c* axis (right).

BiA-I

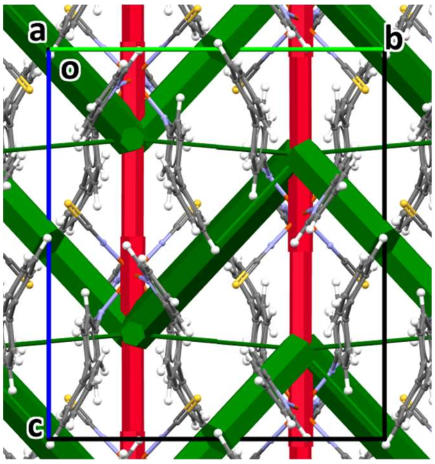

HS−>LS interaction energy changes viewed along **a** axis between alternating layers.

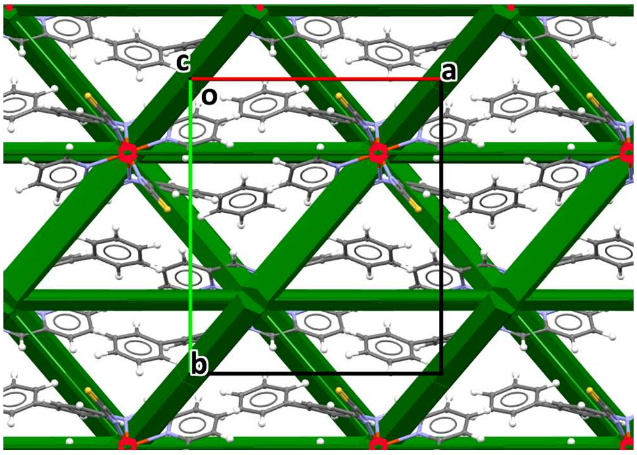

HS−>LS interaction energy changes viewed along **c** axis down multiple layers.

BiA-II

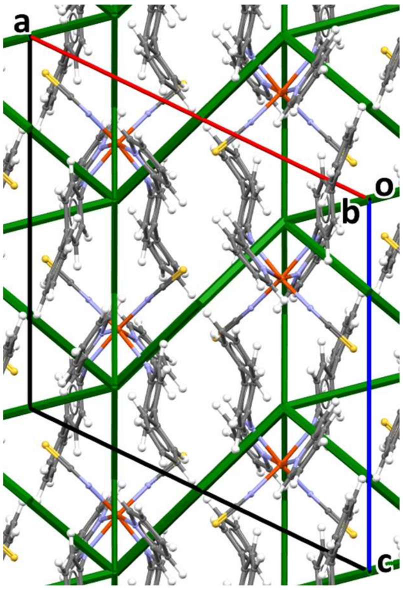

HS−>LS interaction energy changes viewed along **b** axis between alternating layers.

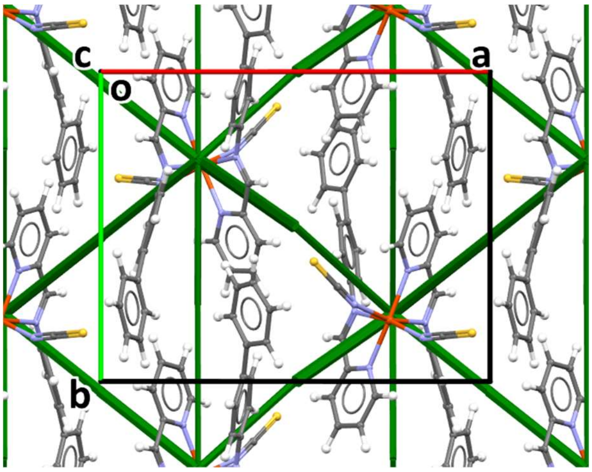

HS−>LS interaction energy changes viewed along **c** axis down multiple layers.

Figure S7: Energy difference frameworks for polymorphs of $Fe(PM\text{-}BiA)_2(NCS)_2$. For clarity, struts are only shown for the intermolecular first molecular coordination sphere (i.e. first nearest neighbours) where the interaction energy changes by more than 2.5 kJ mol^{-1}.

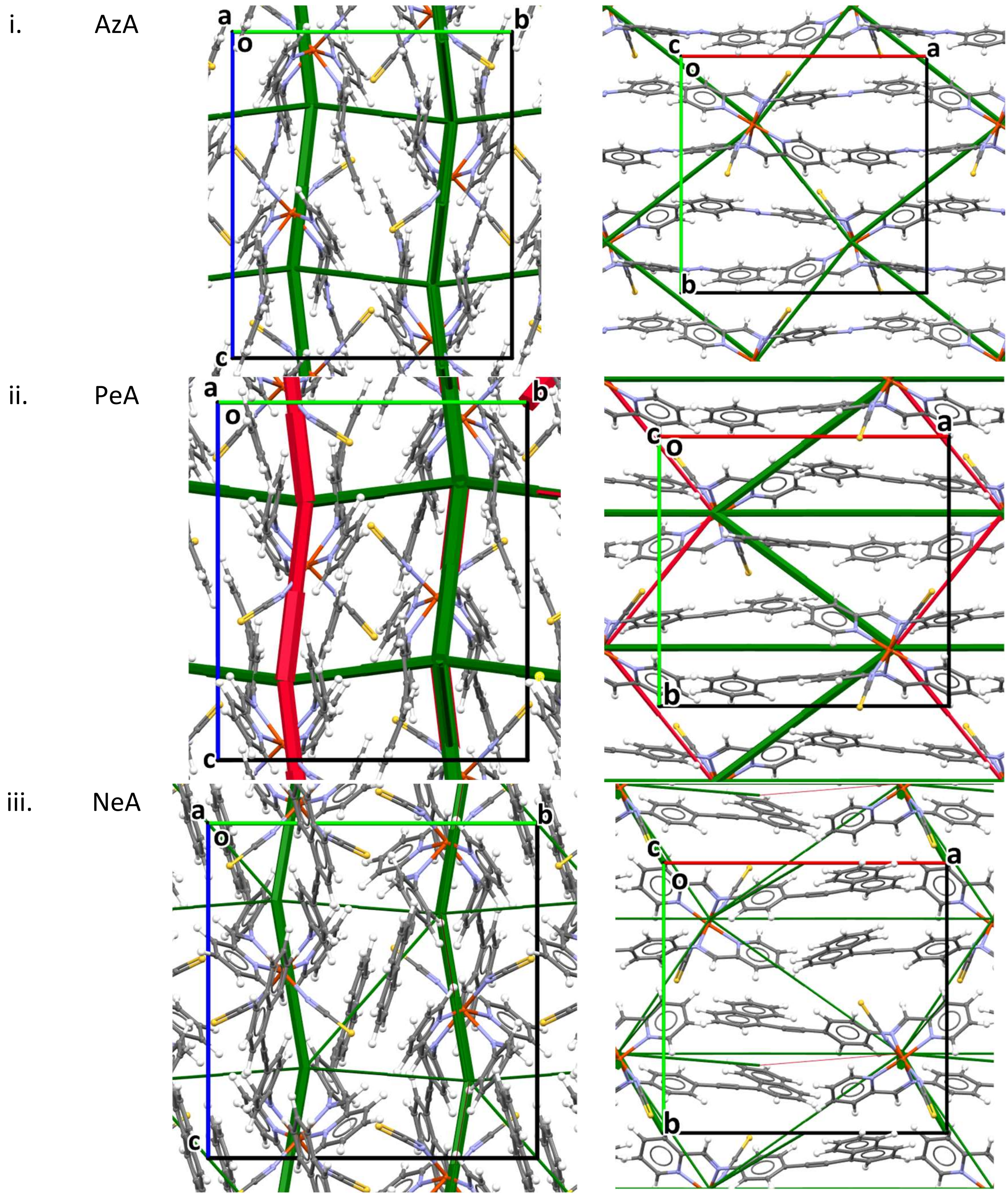

Figure S8: Energy difference framework for (i) $Fe(PM\text{-}AzA)_2(NCS)_2$, (ii) $Fe(PM\text{-}PeA)_2(NCS)_2$ and (iii) $Fe(PM\text{-}NeA)_2(NCS)_2$. The viewing directions are along the a (left) and c (right) axes.

i.

ii.

iii.

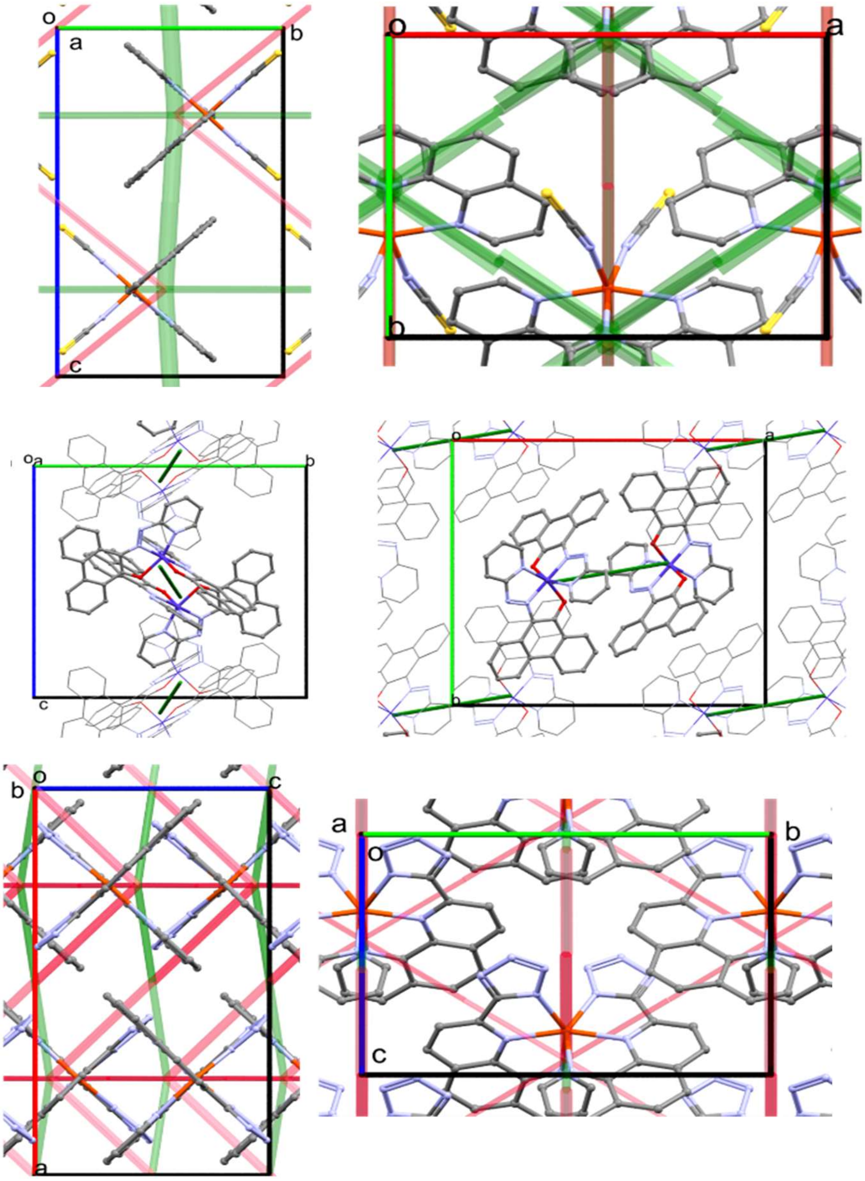

Figure S9: Energy difference frameworks for **i.** $Fe(phen)_2(NCS)_2$, **ii.** bis(10-((pyridine-2-yl)diazenyl)phenanthrene-9-olato)-cobalt and **iii.** $Fe(phen\text{-}Tetrazol)_2$.

Section S6: Data used for generation of energy difference frameworks.

Table S6: Interaction−energy changes (in kJ mol^{-1}) in the HS → LS transition of $Fe(PM\text{-}L)_2(NCS)_2$ structures. The figures in the top and bottom rows are plotted in Fig.7 in the main text.

**Parameter**	**Structure**				
	**BiA-I**	**PeA**	**AzA**	**BiA-II**	**NeA**
**ΔT_{60} (K)**	5.0	14.0	60.0	81.0	97.0
**Interaction Energy Changes ΔE_{TOT} (kJ mol^{-1})**					
**A**	14.6	9.6	−8.8	−1.3	−6.9
**B**	13.0	12.1	−8.8	−1.3	−6.9
**C**	−14.3	−5.1	−0.6	−3.0	−1.7
**D**	−14.3	−5.1	−0.6	−3.0	−1.7
**E**	−3.7	−10.0	−3.6	−2.9	−2.0
**F**	−3.3	−10.2	−3.6	−2.9	−2.0
**G**	−3.3	−4.9	−3.9	−3.7	0.5
**H**	−3.3	−4.8	−3.9	−3.7	0.5
**I**	−2.7	12.8	1.0	−6.6	−4.9
**J**	−2.3	−0.3	−1.6	−5.9	−1.4
**K**	−2.4	2.9	−3.2	−5.9	−1.8
**L**	−2.4	3.6	−3.2	−5.9	−1.8
**M**	−23.0	−6.5	−4.1	0.6	−1.9
**N**	−22.9	−7.2	−4.1	0.6	−1.9
**O**	−2.8	−21.0	−0.1	−4.4	−2.1
**∑Δ|E_{Tot}|**	128.3	116.1	51.1	51.7	38.0

Table S7: PIXEL-C interaction energy changes (HS>LS) for first intermolecular co-ordination sphere contacts of $Fe(PM\text{-}L)_2(NCS)_2$ complexes and other structures studied.

**BiA-I**	**State**	**Interaction Transformation Matrix and Vector**												**Centroid Distances**	$\boldsymbol{E}_{\mathbf{Elec}}$	$\boldsymbol{E}_{\mathbf{Pol}}$	$\boldsymbol{E}_{\mathbf{Disp}}$	$\boldsymbol{E}_{\mathbf{Rep}}$	$\boldsymbol{E}_{\mathbf{Tot}}$
														(Å)			kJ mol^{-1}		
**A**	HS	1	0	0	0	−1	0	0	0	1	0	0.5	−0.5	8.804	−58.8	−28.1	−80.5	59.2	−108.1
	LS	1	0	0	0	−1	0	0	0	1	0	0.5	−0.5	9.140	−49.0	−28.9	−72.6	57.0	−93.5
	LS−HS													0.336	9.8	−0.8	7.9	−2.2	14.6
**B**	HS	1	0	0	0	−1	0	0	0	1	0	0.5	0.5	8.805	−58.8	−28.1	−80.5	61.1	−106.2
	LS	1	0	0	0	−1	0	0	0	1	0	0.5	0.5	9.141	−49.0	−28.9	−72.6	57.3	−93.2
	LS−HS													0.336	9.8	−0.8	7.9	−3.8	13.0
**C**	HS	1	0	0	0	1	0	0	0	1	−1	0	0	12.949	14.2	−4.9	−33.4	10.3	−13.8
	LS	1	0	0	0	1	0	0	0	1	−1	0	0	12.370	9.4	−7.4	−57.9	27.7	−28.1
	LS−HS													−0.579	−4.8	−2.5	−24.5	17.4	−14.3
**D**	HS	1	0	0	0	1	0	0	0	1	1	0	0	12.949	14.2	−4.9	−33.4	10.3	−13.8
	LS	1	0	0	0	1	0	0	0	1	1	0	0	12.370	9.4	−7.4	−57.9	27.7	−28.1
														−0.579	−4.8	−2.5	−24.5	17.4	−14.3
**E**	HS	1	0	0	0	−1	0	0	0	1	−1	0.5	0.5	15.659	−7.4	−5.5	−12.3	7.9	−17.2
	LS	1	0	0	0	−1	0	0	0	1	−1	0.5	0.5	15.381	−11.3	−8.6	−15.6	14.6	−20.9
	LS−HS													−0.278	−3.9	−3.1	−3.3	6.7	−3.7
**F**	HS	1	0	0	0	−1	0	0	0	1	1	0.5	0.5	15.659	−7.4	−5.5	−12.3	7.9	−17.2
	LS	1	0	0	0	−1	0	0	0	1	1	0.5	0.5	15.381	−11.1	−8.4	−15.5	14.6	−20.5
	LS−HS													−0.278	−3.7	−2.9	−3.2	6.7	−3.3
**G**	HS	1	0	0	0	−1	0	0	0	1	−1	0.5	−0.5	15.659	−7.4	−5.5	−12.3	8.0	−17.2
	LS	1	0	0	0	−1	0	0	0	1	−1	0.5	−0.5	15.381	−11.1	−8.4	−15.5	14.5	−20.5
	LS−HS													−0.278	−3.7	−2.9	−3.2	6.5	−3.3
**H**	HS	1	0	0	0	−1	0	0	0	1	1	0.5	−0.5	15.659	−7.4	−5.5	−12.3	7.9	−17.2
	LS	1	0	0	0	−1	0	0	0	1	1	0.5	−0.5	15.381	−11.1	−8.4	−15.5	14.5	−20.5
														−0.278	−3.7	−2.9	−3.2	6.6	−3.3
**I**	HS	−1	0	0	0	1	0	0	0	−1	2	−0.5	0.5	10.000	−19.4	−11.2	−48.3	30.0	−48.9
	LS	−1	0	0	0	1	0	0	0	−1	2	−0.5	0.5	9.703	−21.6	−14.8	−53.3	38.2	−51.6

BiA-I	State	Interaction Transformation Matrix and Vector												Centroid Distances (Å)	E_{Elec}	E_{Pol}	E_{Disp} kJ mol⁻¹	E_{Rep}	E_{Tot}
	LS–HS													−0.297	−2.2	−3.6	−5.0	8.2	−2.7
**J**	HS	−1	0	0	0	1	0	0	0	−1	1	0.5	0.5	10.000	−19.4	−11.2	−48.3	30.0	−48.9
	LS	−1	0	0	0	1	0	0	0	−1	1	0.5	0.5	9.703	−21.6	−14.9	−53.3	38.2	−51.5
	LS–HS													−0.297	−2.2	−3.7	−5.0	8.6	−2.3
**K**	HS	−1	0	0	0	1	0	0	0	−1	1	−0.5	0.5	10.000	−19.4	−11.2	−48.3	30.1	−48.8
	LS	−1	0	0	0	1	0	0	0	−1	1	−0.5	0.5	9.703	−21.6	−14.9	−53.3	38.6	−51.2
	LS–HS													−0.297	−2.2	−3.6	−5.0	8.4	−2.4
**L**	HS	−1	0	0	0	1	0	0	0	−1	2	0.5	0.5	10.000	−19.4	−11.2	−48.3	30.1	−48.8
	LS	−1	0	0	0	1	0	0	0	−1	2	0.5	0.5	9.703	−21.6	−14.8	−53.3	38.5	−51.2
														−0.297	−2.2	−3.6	−5.0	8.4	−2.4
**M**	HS	−1	0	0	0	−1	0	0	0	−1	1	0	0	12.870	−27.8	−3.2	−4.1	0.2	−34.9
	LS	−1	0	0	0	−1	0	0	0	−1	1	0	0	12.494	−45.3	−10.4	−12.5	10.3	−57.9
	LS–HS													−0.376	−17.5	−7.2	−8.4	10.1	−23.0
**N**	HS	−1	0	0	0	−1	0	0	0	−1	2	1	0	12.870	−27.8	−3.2	−4.1	0.2	−34.9
	LS	−1	0	0	0	−1	0	0	0	−1	2	1	0	12.494	−45.1	−10.2	−12.7	10.3	−57.8
	LS–HS													−0.376	−17.3	−7.0	−8.6	10.1	−22.9
**O**	HS	−1	0	0	0	−1	0	0	0	−1	2	0	0	12.870	−23.7	−16.6	−28.4	30.9	−37.8
	LS	−1	0	0	0	−1	0	0	0	−1	2	0	0	12.494	−28.6	−16.3	−25.6	29.9	−40.6
	LS–HS													−0.376	−4.9	0.3	2.8	−1.0	−2.8

PeA	State	Interaction Transformation Matrix and Vector												Centroid Distances	E_{Elec}	E_{Pol}	E_{Disp}	E_{Rep}	E_{Tot}
														(Å)			kJ mol^{-1}		
**A**	HS	1	0	0	0	−1	0	0	0	1	0	1.5	−0.5	8.477	−57.5	−30.5	−76.6	66.5	−98.1
	LS	1	0	0	0	−1	0	0	0	1	0	1.5	−0.5	8.724	−53.1	−33.7	−71.9	70.1	−88.5
	LS−HS													0.247	4.4	−3.2	4.7	3.6	9.6
**B**	HS	1	0	0	0	−1	0	0	0	1	0	1.5	0.5	8.477	−57.5	−30.5	−76.6	66.5	−98.1
	LS	1	0	0	0	−1	0	0	0	1	0	1.5	0.5	8.724	−53.1	−33.7	−71.9	72.7	−86.0
	LS−HS													0.247	4.4	−3.2	4.7	6.2	12.1
**C**	HS	1	0	0	0	1	0	0	0	1	−1	0	0	15.657	6.5	−3.7	−32.2	12.4	−17.1
	LS	1	0	0	0	1	0	0	0	1	−1	0	0	14.357	8.2	−4.7	−46.7	21.1	−22.2
	LS−HS													−1.300	1.7	−1.0	−14.5	8.7	−5.1
**D**	HS	1	0	0	0	1	0	0	0	1	1	0	0	15.657	6.5	−3.7	−32.2	12.4	−17.1
	LS	1	0	0	0	1	0	0	0	1	1	0	0	14.357	8.2	−4.7	−46.7	21.1	−22.2
														−1.300	1.7	−1.0	−14.5	8.7	−5.1
**E**	HS	1	0	0	0	−1	0	0	0	1	−1	1.5	0.5	18.193	−9.3	−5.6	−14.7	11.1	−18.5
	LS	1	0	0	0	−1	0	0	0	1	−1	1.5	0.5	16.800	−10.1	−9.6	−29.9	21.1	−28.5
	LS−HS													−1.393	−0.8	−4.0	−15.2	10.0	−10.0
**F**	HS	1	0	0	0	−1	0	0	0	1	1	1.5	−0.5	18.193	−9.3	−5.6	−14.7	11.1	−18.5
	LS	1	0	0	0	−1	0	0	0	1	1	1.5	−0.5	16.800	−10.1	−9.6	−29.9	21.0	−28.7
	LS−HS													−1.393	−0.8	−4.0	−15.2	9.9	−10.2
**G**	HS	1	0	0	0	−1	0	0	0	1	−1	1.5	−0.5	17.408	−6.5	−7.9	−26.3	16.1	−24.6
	LS	1	0	0	0	−1	0	0	0	1	−1	1.5	−0.5	16.800	−10.2	−9.6	−30.5	20.8	−29.5
	LS−HS													−0.608	−3.7	−1.7	−4.2	4.7	−4.9
**H**	HS	1	0	0	0	−1	0	0	0	1	1	1.5	0.5	17.408	−6.5	−7.9	−26.3	16.1	−24.6
	LS	1	0	0	0	−1	0	0	0	1	1	1.5	0.5	16.800	−10.2	−9.6	−30.5	21.0	−29.4
														−0.608	−3.7	−1.7	−4.2	4.9	−4.8
**I**	HS	−1	0	0	0	−1	0	0	0	−1	2	2	0	11.597	−48.6	−19.5	−35.4	28.3	−75.2
	LS	−1	0	0	0	−1	0	0	0	−1	2	2	0	12.255	−42.3	−15.5	−23.6	19.1	−62.4
	LS−HS													0.658	6.3	4.0	11.8	−9.2	12.8
**J**	HS	−1	0	0	0	−1	0	0	0	−1	1	2	0	13.511	−23.5	−11.5	−23.4	16.5	−41.9
	LS	−1	0	0	0	−1	0	0	0	−1	1	2	0	12.255	−30.2	−20.0	−30.5	38.6	−42.2

PeA	State	Interaction Transformation Matrix and Vector												Centroid Distances (Å)	E_{Elec}	E_{Pol}	E_{Disp} kJ mol^{-1}	E_{Rep}	E_{Tot}
	LS−HS													−1.256	−6.7	−8.5	−7.1	22.1	−0.3
**K**	HS	−1	0	0	0	1	0	0	0	−1	2	−0.5	0.5	9.382	−19.5	−11.3	−52.8	31.0	−52.6
	LS	−1	0	0	0	1	0	0	0	−1	2	−0.5	0.5	10.292	−21.6	−13.1	−50.0	35.0	−49.7
	LS−HS													0.910	−2.1	−1.8	2.8	4.0	2.9
**L**	HS	−1	0	0	0	1	0	0	0	−1	2	0.5	0.5	9.382	−19.5	−11.3	−52.8	31.0	−52.6
	LS	−1	0	0	0	1	0	0	0	−1	2	0.5	0.5	10.292	−21.6	−13.1	−50.0	35.7	−49.0
														0.910	−2.1	−1.8	2.8	4.7	3.6
**M**	HS	−1	0	0	0	1	0	0	0	−1	1	−0.5	0.5	12.250	−17.8	−10.1	−41.7	27.6	−42.0
	LS	−1	0	0	0	1	0	0	0	−1	1	−0.5	0.5	10.292	−21.5	−13.1	−49.7	35.7	−48.5
	LS−HS													−1.958	−3.7	−3.0	−8.0	8.1	−6.5
**N**	HS	−1	0	0	0	1	0	0	0	−1	1	0.5	0.5	12.250	−17.8	−10.1	−41.7	27.6	−42.0
	LS	−1	0	0	0	1	0	0	0	−1	1	0.5	0.5	10.292	−21.5	−13.1	−49.7	35.0	−49.2
	LS−HS													−1.958	−3.7	−3.0	−8.0	7.4	−7.2
**O**	HS	−1	0	0	0	−1	0	0	0	−1	2	1	0	12.766	−9.4	−11.3	−22.1	21.8	−21.0
	LS	−1	0	0	0	−1	0	0	0	−1	2	1	0	12.255	−29.7	−20.0	−31.0	38.7	−42.0
	LS−HS													−0.511	−20.3	−8.7	−8.9	16.9	−21.0

AzA	State	Interaction Transformation Matrix and Vector												Centroid Distances (Å)	E_{Elec}	E_{Pol}	E_{Disp} kJ mol^{-1}	E_{Rep}	E_{Tot}
**A**	HS	1	0	0	0	−1	0	0	0	1	0	0.5	−0.5	8.569	−59.8	−31.4	−76.5	68.4	−99.4
	LS	1	0	0	0	−1	0	0	0	1	0	0.5	−0.5	8.368	−71.7	−38.0	−84.3	85.8	−108.2
	LS−HS													−0.201	−11.9	−6.6	−7.8	17.4	−8.8
**B**	HS	1	0	0	0	−1	0	0	0	1	0	0.5	0.5	8.569	−59.8	−31.4	−76.5	68.4	−99.4
	LS	1	0	0	0	−1	0	0	0	1	0	0.5	0.5	8.368	−71.7	−38.0	−84.3	85.8	−108.2
	LS−HS													−0.201	−11.9	−6.6	−7.8	17.4	−8.8
**C**	HS	1	0	0	0	1	0	0	0	1	−1	0	0	15.121	4.3	−3.5	−38.0	13.7	−23.5
	LS	1	0	0	0	1	0	0	0	1	−1	0	0	14.998	3.4	−4.6	−43.7	20.7	−24.1
	LS−HS													−0.123	−0.9	−1.1	−5.7	7.0	−0.6
**D**	HS	1	0	0	0	1	0	0	0	1	1	0	0	15.121	4.3	−3.5	−38.0	13.7	−23.5
	LS	1	0	0	0	1	0	0	0	1	1	0	0	14.998	3.4	−4.6	−43.7	20.7	−24.1
														−0.123	−0.9	−1.1	−5.7	7.0	−0.6
**E**	HS	1	0	0	0	−1	0	0	0	1	1	0.5	−0.5	17.759	−8.4	−5.5	−13.7	11.4	−16.2
	LS	1	0	0	0	−1	0	0	0	1	1	0.5	−0.5	17.529	−11.7	−6.8	−16.3	15.1	−19.8
	LS−HS													−0.230	−3.3	−1.3	−2.6	3.7	−3.6
**F**	HS	1	0	0	0	−1	0	0	0	1	−1	0.5	0.5	17.759	−8.4	−5.5	−13.7	11.4	−16.2
	LS	1	0	0	0	−1	0	0	0	1	−1	0.5	0.5	17.529	−11.7	−6.8	−16.3	15.1	−19.8
	LS−HS													−0.230	−3.3	−1.3	−2.6	3.7	−3.6
**G**	HS	1	0	0	0	−1	0	0	0	1	−1	0.5	−0.5	16.995	−5.7	−7.7	−23.4	15.0	−21.8
	LS	1	0	0	0	−1	0	0	0	1	−1	0.5	−0.5	16.813	−8.4	−9.9	−28.1	20.7	−25.7
	LS−HS													−0.182	−2.7	−2.2	−4.7	5.7	−3.9
**H**	HS	1	0	0	0	−1	0	0	0	1	1	0.5	0.5	16.995	−5.7	−7.7	−23.4	15.0	−21.8
	LS	1	0	0	0	−1	0	0	0	1	1	0.5	0.5	16.813	−8.4	−9.9	−28.1	20.7	−25.7
														−0.182	−2.7	−2.2	−4.7	5.7	−3.9
**I**	HS	−1	0	0	0	−1	0	0	0	−1	0	1	0	13.315	−30.5	−14.9	−28.9	28.3	−46.0
	LS	−1	0	0	0	−1	0	0	0	−1	0	1	0	13.037	−30.2	−16.9	−31.5	33.6	−45.0
	LS−HS													−0.278	0.3	−2.0	−2.6	5.3	1.0
**J**	HS	−1	0	0	0	−1	0	0	0	−1	1	1	0	11.657	−55.3	−22.8	−35.2	38.0	−75.4
	LS	−1	0	0	0	−1	0	0	0	−1	1	1	0	11.431	−55.8	−24.8	−40.5	44.1	−77.0

AzA	State	Interaction Transformation Matrix and Vector												Centroid Distances (Å)	E_{Elec}	E_{Pol}	E_{Disp}	E_{Rep}	E_{Tot}
																	kJ mol^{-1}		
	LS−HS													−0.226	−0.5	−2.0	−5.3	6.1	−1.6
**K**	HS	−1	0	0	0	1	0	0	0	−1	1	−0.5	0.5	9.319	−17.7	−12.3	−54.2	32.0	−52.2
	LS	−1	0	0	0	1	0	0	0	−1	1	−0.5	0.5	9.270	−23.4	−15.4	−56.9	40.2	−55.4
	LS−HS													−0.049	−5.7	−3.1	−2.7	8.2	−3.2
**L**	HS	−1	0	0	0	1	0	0	0	−1	1	0.5	0.5	9.319	−17.7	−12.3	−54.2	32.0	−52.2
	LS	−1	0	0	0	1	0	0	0	−1	1	0.5	0.5	9.270	−23.4	−15.4	−56.9	40.3	−55.4
														−0.049	−5.7	−3.1	−2.7	8.3	−3.2
**M**	HS	−1	0	0	0	1	0	0	0	−1	0	−0.5	0.5	11.896	−14.4	−9.3	−42.2	23.2	−42.7
	LS	−1	0	0	0	1	0	0	0	−1	0	−0.5	0.5	11.726	−20.3	−11.7	−49.1	34.3	−46.8
	LS−HS													−0.170	−5.9	−2.4	−6.9	11.1	−4.1
**N**	HS	−1	0	0	0	1	0	0	0	−1	0	0.5	0.5	11.896	−14.4	−9.3	−42.2	23.2	−42.7
	LS	−1	0	0	0	1	0	0	0	−1	0	0.5	0.5	11.726	−20.3	−11.7	−49.1	34.4	−46.8
	LS−HS													−0.170	−5.9	−2.4	−6.9	11.2	−4.1
**O**	HS	−1	0	0	0	−1	0	0	0	−1	1	0	0	12.825	−8.3	−11.3	−21.9	21.5	−20.0
	LS	−1	0	0	0	−1	0	0	0	−1	1	0	0	12.530	−10.9	−12.8	−23.8	27.4	−20.1
	LS−HS													−0.295	−2.6	−1.5	−1.9	5.9	−0.1

BiA-II	State	Interaction Transformation Matrix and Vector												Centroid Distances	E_{Elec}	E_{Pol}	E_{Disp}	E_{Rep}	E_{Tot}
														(Å)	kJ mol^{-1}				
**A**	HS	1	0	0	0	−1	0	0	0	1	0	0.5	−0.5	8.719	−57.4	−28.8	−80.4	68.6	−97.9
	LS	1	0	0	0	−1	0	0	0	1	0	0.5	−0.5	8.551	−61.0	−34.4	−88.0	84.2	−99.2
	LS−HS													−0.168	−3.6	−5.6	−7.6	15.6	−1.3
**B**	HS	1	0	0	0	−1	0	0	0	1	0	0.5	0.5	8.719	−57.4	−28.8	−80.4	68.6	−97.9
	LS	1	0	0	0	−1	0	0	0	1	0	0.5	0.5	8.551	−61.0	−34.4	−88.0	84.2	−99.2
	LS−HS													−0.168	−3.6	−5.6	−7.6	15.6	−1.3
**C**	HS	1	0	0	0	1	0	0	0	1	0	−1	0	12.602	9.6	−5.9	−44.2	19.6	−20.9
	LS	1	0	0	0	1	0	0	0	1	0	−1	0	12.362	9.6	−6.8	−51.3	24.6	−23.9
	LS−HS													−0.240	0.0	−0.9	−7.1	5.0	−3.0
**D**	HS	1	0	0	0	1	0	0	0	1	0	1	0	12.602	9.6	−5.9	−44.2	19.6	−20.9
	LS	1	0	0	0	1	0	0	0	1	0	1	0	12.362	9.6	−6.8	−51.3	24.6	−23.9
														−0.240	0.0	−0.9	−7.1	5.0	−3.0
**E**	HS	1	0	0	0	−1	0	0	0	1	0	1.5	−0.5	15.996	−6.5	−5.0	−11.5	8.4	−14.6
	LS	1	0	0	0	−1	0	0	0	1	0	1.5	−0.5	15.568	−8.6	−6.1	−13.8	11.0	−17.5
	LS−HS													−0.428	−2.1	−1.1	−2.3	2.6	−2.9
**F**	HS	1	0	0	0	−1	0	0	0	1	0	1.5	0.5	15.996	−6.5	−5.0	−11.5	8.4	−14.6
	LS	1	0	0	0	−1	0	0	0	1	0	1.5	0.5	15.568	−8.6	−6.1	−13.8	11.0	−17.5
	LS−HS													−0.428	−2.1	−1.1	−2.3	2.6	−2.9
**G**	HS	1	0	0	0	−1	0	0	0	1	0	−0.5	−0.5	14.621	−9.1	−7.2	−18.8	12.6	−22.6
	LS	1	0	0	0	−1	0	0	0	1	0	−0.5	−0.5	14.475	−10.3	−9.4	−22.8	16.2	−26.3
	LS−HS													−0.146	−1.2	−2.2	−4.0	3.6	−3.7
**H**	HS	1	0	0	0	−1	0	0	0	1	0	−0.5	0.5	14.621	−9.1	−7.2	−18.8	12.6	−22.6
	LS	1	0	0	0	−1	0	0	0	1	0	−0.5	0.5	14.475	−10.3	−9.4	−22.8	16.2	−26.3
														−0.146	−1.2	−2.2	−4.0	3.6	−3.7
**I**	HS	−1	0	0	0	−1	0	0	0	−1	1	0	1	12.318	−39.0	−8.0	−12.6	7.9	−51.7
	LS	−1	0	0	0	−1	0	0	0	−1	1	0	1	12.091	−46.8	−12.1	−15.5	16.0	−58.3
	LS−HS													−0.227	−7.8	−4.1	−2.9	8.1	−6.6

**J**	HS	−1	0	0	0	−1	0	0	0	−1	0	0	0	9.833	−27.7	−14.7	−77.7	39.4	−80.7
	LS	−1	0	0	0	−1	0	0	0	−1	0	0	0	9.750	−35.3	−19.5	−86.7	54.8	−86.6
	LS−HS													−0.083	−7.6	−4.8	−9.0	15.4	−5.9
**K**	HS	−1	0	0	0	1	0	0	0	−1	0	0.5	0.5	12.090	−34.2	−12.0	−21.3	20.1	−47.5
	LS	−1	0	0	0	1	0	0	0	−1	0	0.5	0.5	11.774	−42.0	−17.2	−26.4	32.2	−53.4
	LS−HS													−0.316	−7.8	−5.2	−5.1	12.1	−5.9
**L**	HS	−1	0	0	0	1	0	0	0	−1	0	−0.5	0.5	12.090	−34.2	−12.0	−21.3	20.1	−47.5
	LS	−1	0	0	0	1	0	0	0	−1	0	−0.5	0.5	11.774	−42.0	−17.2	−26.4	32.2	−53.4
														−0.316	−7.8	−5.2	−5.1	12.1	−5.9
**M**	HS	−1	0	0	0	1	0	0	0	−1	1	−0.5	0.5	10.171	−17.2	−11.1	−44.6	28.2	−44.7
	LS	−1	0	0	0	1	0	0	0	−1	1	−0.5	0.5	10.090	−19.2	−13.2	−44.8	33.0	−44.1
	LS−HS													−0.081	−2.0	−2.1	−0.2	4.8	0.6
**N**	HS	−1	0	0	0	1	0	0	0	−1	1	0.5	0.5	10.171	−17.2	−11.1	−44.6	28.2	−44.7
	LS	−1	0	0	0	1	0	0	0	−1	1	0.5	0.5	10.090	−19.2	−13.2	−44.8	33.0	−44.1
	LS−HS													−0.081	−2.0	−2.1	−0.2	4.8	0.6
**O**	HS	−1	0	0	0	−1	0	0	0	−1	1	1	1	13.145	−14.8	−12.6	−23.5	23.5	−27.4
	LS	−1	0	0	0	−1	0	0	0	−1	1	1	1	12.752	−22.0	−15.3	−25.6	31.0	−31.8
	LS−HS													−0.393	−7.2	−2.7	−2.1	7.5	−4.4

NeA	State	Interaction Transformation Matrix and Vector												Centroid Distances (Å)	E_{Elec}	E_{Pol}	E_{Disp}	E_{Rep}	E_{Tot}
																	kJ mol^{-1}		
**A**	HS	1	0	0	0	−1	0	0	0	1	0	0.5	−0.5	8.124	−52.7	−27.4	−90.5	64.6	−106.0
	LS	1	0	0	0	−1	0	0	0	1	0	0.5	−0.5	7.878	−61.7	−34.0	−98.6	81.4	−112.9
	LS−HS													−0.246	−9.0	−6.6	−8.1	16.8	−6.9
**B**	HS	1	0	0	0	−1	0	0	0	1	0	0.5	0.5	8.124	−52.7	−27.4	−90.5	64.6	−106.0
	LS	1	0	0	0	−1	0	0	0	1	0	0.5	0.5	7.878	−61.7	−34.0	−98.6	81.4	−112.9
	LS−HS													−0.246	−9.0	−6.6	−8.1	16.8	−6.9
**C**	HS	1	0	0	0	1	0	0	0	1	−1	0	0	16.489	6.3	−3.8	−51.0	16.3	−32.3
	LS	1	0	0	0	1	0	0	0	1	−1	0	0	16.510	4.6	−4.7	−58.5	24.7	−34.0
	LS−HS													0.021	−1.7	−0.9	−7.5	8.4	−1.7
**D**	HS	1	0	0	0	1	0	0	0	1	1	0	0	16.489	6.3	−3.8	−51.0	16.3	−32.3
	LS	1	0	0	0	1	0	0	0	1	1	0	0	16.510	4.5	−4.7	−58.5	24.7	−34.0
														0.021	−1.8	−0.9	−7.5	8.4	−1.7
**E**	HS	1	0	0	0	−1	0	0	0	1	−1	0.5	0.5	18.419	−10.6	−6.6	−23.4	15.7	−24.8
	LS	1	0	0	0	−1	0	0	0	1	−1	0.5	0.5	18.281	−12.3	−8.3	−27.5	21.3	−26.8
	LS−HS													−0.138	−1.7	−1.7	−4.1	5.6	−2.0
**F**	HS	1	0	0	0	−1	0	0	0	1	1	0.5	−0.5	18.419	−10.6	−6.6	−23.4	15.7	−24.8
	LS	1	0	0	0	−1	0	0	0	1	1	0.5	−0.5	18.281	−12.3	−8.3	−27.5	21.3	−26.8
	LS−HS													−0.138	−1.7	−1.7	−4.1	5.6	−2.0
**G**	HS	1	0	0	0	−1	0	0	0	1	−1	0.5	−0.5	18.344	−13.1	−8.3	−42.3	25.8	−37.9
	LS	1	0	0	0	−1	0	0	0	1	−1	0.5	−0.5	18.305	−15.7	−9.9	−45.9	34.0	−37.4
	LS−HS													−0.039	−2.6	−1.6	−3.6	8.2	0.5
**H**	HS	1	0	0	0	−1	0	0	0	1	1	0.5	0.5	18.344	−13.1	−8.3	−42.3	25.8	−37.9
	LS	1	0	0	0	−1	0	0	0	1	1	0.5	0.5	18.305	−15.7	−9.9	−45.9	34.0	−37.4
														−0.039	−2.6	−1.6	−3.6	8.2	0.5
**I**	HS	−1	0	0	0	−1	0	0	0	−1	0	1	1	13.742	−12.8	−12.0	−24.5	27.8	−21.6
	LS	−1	0	0	0	−1	0	0	0	−1	0	1	1	13.452	−17.4	−14.8	−26.7	32.3	−26.5
	LS−HS													−0.290	−4.6	−2.8	−2.2	4.5	−4.9
**J**	HS	−1	0	0	0	−1	0	0	0	−1	0	0	1	12.075	−39.4	−14.8	−37.9	22.4	−69.8
	LS	−1	0	0	0	−1	0	0	0	−1	0	0	1	11.899	−46.0	−18.2	−39.3	32.3	−71.2

NeA	State	Interaction Transformation Matrix and Vector												Centroid Distances (Å)	E_{Elec}	E_{Pol}	E_{Disp} kJ mol^{-1}	E_{Rep}	E_{Tot}
	LS−HS													−0.176	−6.6	−3.4	−1.4	9.9	−1.4
**K**	HS	−1	0	0	0	1	0	0	0	−1	1	−0.5	0.5	13.646	−21.6	−12.5	−49.2	35.3	−48.0
	LS	−1	0	0	0	1	0	0	0	−1	1	−0.5	0.5	13.653	−26.2	−17.3	−55.4	49.1	−49.8
	LS−HS													0.007	−4.6	−4.8	−6.2	13.8	−1.8
**L**	HS	−1	0	0	0	1	0	0	0	−1	1	0.5	0.5	13.646	−21.6	−12.5	−49.2	35.3	−48.0
	LS	−1	0	0	0	1	0	0	0	−1	1	0.5	0.5	13.653	−26.2	−17.3	−55.4	49.1	−49.8
														0.007	−4.6	−4.8	−6.2	13.8	−1.8
**M**	HS	−1	0	0	0	1	0	0	0	−1	0	−0.5	0.5	9.537	−4.5	−9.8	−44.8	20.0	−39.1
	LS	−1	0	0	0	1	0	0	0	−1	0	−0.5	0.5	9.432	−5.9	−11.4	−47.3	23.5	−41.0
	LS−HS													−0.105	−1.4	−1.6	−2.5	3.5	−1.9
**N**	HS	−1	0	0	0	1	0	0	0	−1	0	0.5	0.5	9.537	−4.5	−9.8	−44.8	20.0	−39.1
	LS	−1	0	0	0	1	0	0	0	−1	0	0.5	0.5	9.432	−5.9	−11.4	−47.3	23.5	−41.0
	LS−HS													−0.105	−1.4	−1.6	−2.5	3.5	−1.9
**O**	HS	−1	0	0	0	−1	0	0	0	−1	1	0	0	14.889	−5.7	−4.0	−45.6	23.7	−31.6
	LS	−1	0	0	0	−1	0	0	0	−1	1	0	0	14.767	−9.1	−6.5	−55.2	37.0	−33.7
	LS−HS													−0.122	−3.4	−2.5	−9.6	13.3	−2.1

Table S8: Correlations for PIXEL energy terms with respect to ΔT_{60} for PM-L Structures studied. Note that the magnitude of **all energies** is not equivalent to the magnitude of **all total energies** because total energies take into account the sign of each energy term, where the magnitude of all energies is from absolute values (see Equation 1).

$$|\Delta E| = |\Delta E_{Elec}| + |\Delta E_{Pol}| + |\Delta E_{Disp}| + |\Delta E_{Rep}| \quad \text{Equation 1}$$

**Feature**	**Symbol**	**Correlation co-efficient (with respect to ΔT_{60})**
Number of Electrons	–	0.53
Sum of all centroid-centroid distance changes (Å)	–	0.68
Magnitude of all centroid-centroid Distance changes (Å)	–	−0.68
Magnitude of all energies (kJ mol^{-1})	$\lvert\Delta E\rvert$	−0.87
Magnitude of all electrostatic terms (kJ mol^{-1})	$\lvert\Delta E_{Elec}\rvert$	−0.81
Magnitude of all polarisation terms (kJ mol^{-1})	$\lvert\Delta E_{Pol}\rvert$	−0.61
Magnitude of all dispersion terms (kJ mol^{-1})	$\lvert\Delta E_{Disp}\rvert$	−0.90
Magnitude of all repulsion terms (kJ mol^{-1})	$\lvert\Delta E_{Rep}\rvert$	0.34
Magnitude of all total energy changes (kJ mol^{-1})	$\lvert\Delta E_{Tot}\rvert$	−0.97
Most positive electrostatic term change (kJ mol^{-1})	Max. +ve ΔE_{Elec}	−0.96
Most positive polarisation term change (kJ mol^{-1})	Max. +ve ΔE_{Pol}	−0.69
Most positive dispersion term change (kJ mol^{-1})	Max. +ve ΔE_{Disp}	−0.89
Most positive repulsion term change (kJ mol^{-1})	Max. +ve ΔE_{Rep}	−0.64
Most positive total interaction energy change (kJ mol^{-1})	Max. +ve ΔE_{Tot}	−0.95
Most negative electrostatic term change (kJ mol^{-1})	Max. −ve ΔE_{Elec}	0.94
Most negative polarisation term change (kJ mol^{-1})	Max. −ve E_{Pol}	0.74
Most negative dispersion term change (kJ mol^{-1})	Max. −ve ΔE_{Disp}	0.83
Most negative repulsion term change (kJ mol^{-1})	Max. −ve ΔE_{Rep}	0.85
Most negative total interaction energy change (kJ mol^{-1})	Max. −ve ΔE_{Tot}	0.97
Largest magnitude electrostatic term change (kJ mol^{-1})	Max. $\lvert\Delta E_{Elec}\rvert$	−0.94
Largest magnitude polarisation term change (kJ mol^{-1})	Max. $\lvert\Delta E_{Pol}\rvert$	−0.74
Largest magnitude dispersion term change (kJ mol^{-1})	Max. $\lvert\Delta E_{Disp}\rvert$	−0.83
Largest magnitude repulsion term change (kJ mol^{-1})	Max. $\lvert\Delta E_{Rep}\rvert$	−0.64
Largest magnitude total interaction energy change (kJ mol^{-1})	Max. $\lvert\Delta E_{Tot}\rvert$	−0.97
Sum of all electrostatic term changes (kJ mol^{-1})	$\sum\Delta E_{Elec}$	−0.57
Sum of polarisation term changes (kJ mol^{-1})	$\sum\Delta E_{Pol}$	0.22
Sum of dispersion term changes (kJ mol^{-1})	$\sum\Delta E_{Disp}$	0.41
Sum of repulsion term changes (kJ mol^{-1})	$\sum\Delta E_{Rep}$	0.87
Sum of total interaction energy changes (kJ mol^{-1})	$\sum\Delta E_{Tot}$	0.45

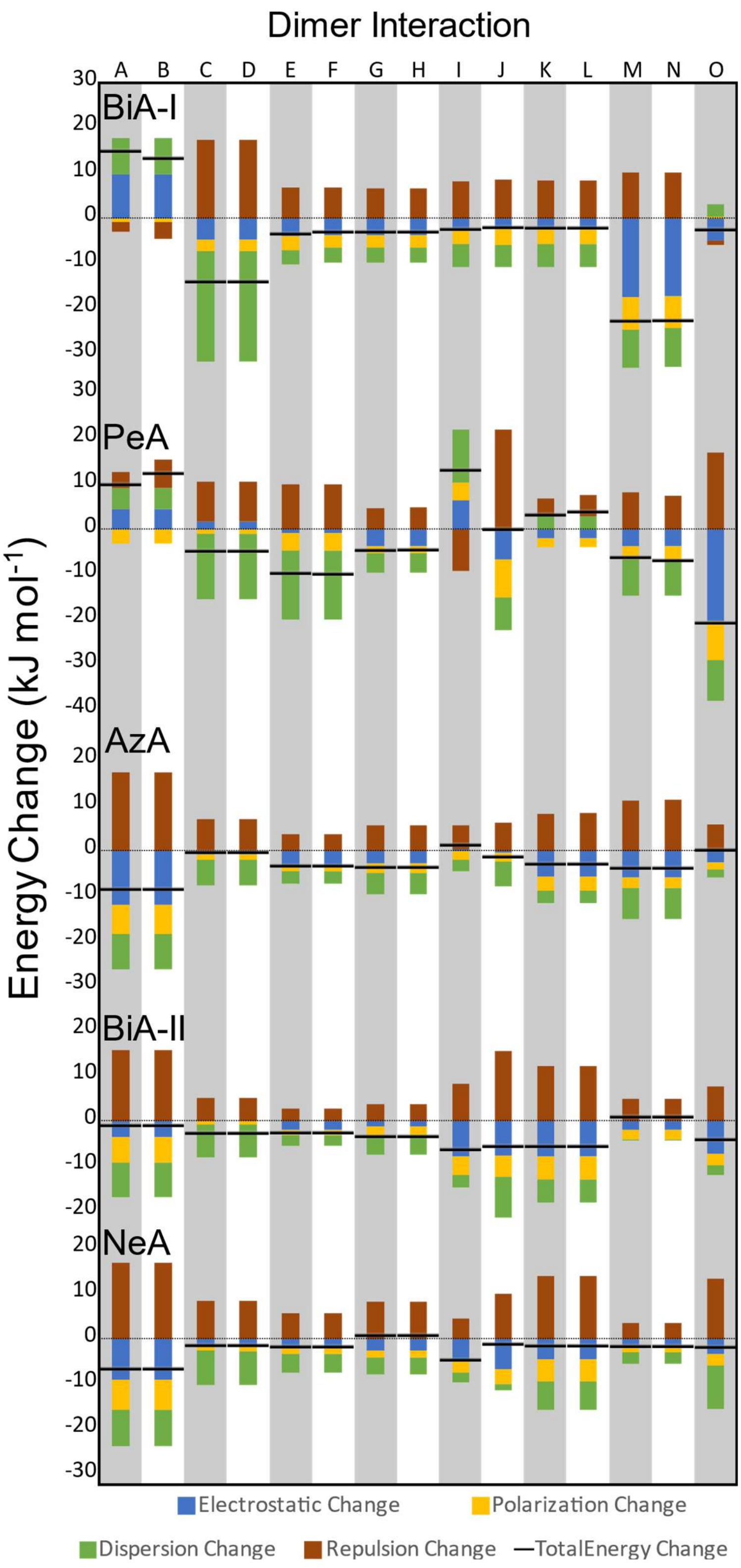

Figure S10: Stacked bar−graph of interaction energy changes from HS to LS structures for PM−L complexes, broken down by separate energy components. The total energy changes are shown as black bars: destabilising changes which appear as pink struts in the framework plots of Figs. 4 and 5 of the main paper are located above the zero-energy; the stabilising changes represented as green struts are below the axis.

Table S9: Interaction Energy changes for some non PM-L complexes. **i.** $Fe(phen)_2(NCS)_2$, (CSD Refcodes HS:KEKVIF, LS:KEKVIF01) **ii.** bis(10-((pyridine-2-yl)diazenyl)phenanthrene-9-olato)-cobalt(ii) (CSD Refcodes HS: PUYROS01, LS: PUYROS), **iii.** $Fe(Phen\text{-}Tetrazol)_2$, (CSD Refcodes HS:QIDJET, LS:QIDJET01).

**i. Phen**	**State**	**Interaction Transformation Matrix and Vector**												**Centroid Distances (Å)**	E_{Elec}	E_{Pol}	E_{Disp} kJ mol^{-1}	E_{Rep}	E_{Tot}
	HS	1	0	0	0	1	0	0	0	−1	0.5	0.5	0.5	8.314	−25.6	−22.2	−68.0	44.5	−71.3
	LS	1	0	0	0	1	0	0	0	−1	0.5	0.5	0.5	8.138	−30.9	−27.8	−74.4	54.4	−78.7
	LS−HS													−0.176	−5.3	−5.6	−6.4	9.9	−7.4
	HS	1	0	0	0	1	0	0	0	−1	−0.5	−0.5	0.5	8.314	−25.6	−22.2	−68.0	44.4	−71.4
	LS	1	0	0	0	1	0	0	0	−1	−0.5	−0.5	0.5	8.138	−30.9	−27.8	−74.4	54.2	−78.9
	LS−HS													−0.176	−5.3	−5.6	−6.4	9.8	−7.5
	HS	1	0	0	0	1	0	0	0	−1	0.5	−0.5	0.5	8.314	−25.6	−22.2	−68.0	44.5	−71.3
	LS	1	0	0	0	1	0	0	0	−1	0.5	−0.5	0.5	8.138	−30.9	−27.8	−74.4	54.2	−78.9
	LS−HS													−0.176	−5.3	−5.6	−6.4	9.7	−7.6
	HS	1	0	0	0	1	0	0	0	−1	−0.5	0.5	0.5	8.314	−25.6	−22.2	−68.0	44.4	−71.4
	LS	1	0	0	0	1	0	0	0	−1	−0.5	0.5	0.5	8.138	−30.9	−27.8	−74.4	54.1	−79.0
														−0.176	−5.3	−5.6	−6.4	9.7	−7.6
	HS	1	0	0	0	−1	0	0	0	1	0	1	−0.5	8.752	−56.0	−23.0	−46.0	32.9	−92.1
	LS	1	0	0	0	−1	0	0	0	1	0	1	−0.5	8.626	−63.0	−27.2	−50.9	38.8	−102.3
	LS−HS													−0.126	−7.0	−4.2	−4.9	5.9	−10.2
	HS	1	0	0	0	−1	0	0	0	1	0	1	0.5	8.752	−56.0	−23.0	−46.0	32.9	−92.1
	LS	1	0	0	0	−1	0	0	0	1	0	1	0.5	8.626	−63.0	−27.2	−50.9	38.8	−102.4
	LS−HS													−0.126	−7.0	−4.2	−4.9	5.9	−10.3
	HS	1	0	0	0	−1	0	0	0	−1	0.5	1.5	0	11.877	−27.9	−6.4	−11.8	12.0	−34.2
	LS	1	0	0	0	−1	0	0	0	−1	0.5	1.5	0	11.639	−31.8	−7.3	−11.8	11.8	−39.2
	LS−HS													−0.238	−3.9	−0.9	0.0	−0.2	−5.0
	HS	1	0	0	0	−1	0	0	0	−1	0.5	1.5	1	11.877	−27.9	−6.4	−11.8	12.0	−34.2
	LS	1	0	0	0	−1	0	0	0	−1	0.5	1.5	1	11.639	−31.8	−7.3	−11.8	11.8	−39.2
														−0.238	−3.9	−0.9	0.0	−0.2	−5.0
	HS	1	0	0	0	−1	0	0	0	−1	−0.5	1.5	0	11.877	−27.9	−6.4	−11.8	12.0	−34.2
	LS	1	0	0	0	−1	0	0	0	−1	−0.5	1.5	0	11.639	−31.8	−7.3	−11.8	11.7	−39.2

**i. Phen**	**State**	**Interaction Transformation Matrix and Vector**												**Centroid Distances**	$\boldsymbol{E}_{\mathbf{Elec}}$	$\boldsymbol{E}_{\mathbf{Pol}}$	$\boldsymbol{E}_{\mathbf{Disp}}$	$\boldsymbol{E}_{\mathbf{Rep}}$	$\boldsymbol{E}_{\mathbf{Tot}}$
														(Å)			kJ mol^{-1}		
	LS−HS													−0.238	−3.9	−0.9	0.0	−0.3	−5.0
	HS	1	0	0	0	−1	0	0	0	−1	−0.5	1.5	1	11.877	−27.9	−6.4	−11.8	12.0	−34.2
	LS	1	0	0	0	−1	0	0	0	−1	−0.5	1.5	1	11.639	−31.8	−7.3	−11.8	11.7	−39.2
	LS−HS													−0.238	−3.9	−0.9	0.0	−0.3	−5.0
	HS	1	0	0	0	1	0	0	0	1	0	−1	0	10.163	−26.9	−7.8	−12.8	6.1	−41.3
	LS	1	0	0	0	1	0	0	0	1	0	−1	0	10.090	−30.5	−9.2	−13.5	7.4	−45.8
	LS−HS													−0.073	−3.6	−1.4	−0.7	1.3	−4.5
	HS	1	0	0	0	1	0	0	0	1	0	1	0	10.163	−26.9	−7.8	−12.8	6.1	−41.3
	LS	1	0	0	0	1	0	0	0	1	0	1	0	10.090	−30.5	−9.2	−13.5	7.4	−45.8
														−0.073	−3.6	−1.4	−0.7	1.3	−4.5
	HS	1	0	0	0	−1	0	0	0	−1	−0.5	0.5	0	12.264	6.2	−2.7	−8.8	4.1	−1.2
	LS	1	0	0	0	−1	0	0	0	−1	−0.5	0.5	0	12.074	7.0	−4.0	−10.9	6.7	−1.2
	LS−HS													−0.190	0.8	−1.3	−2.1	2.6	0.0
	HS	1	0	0	0	−1	0	0	0	−1	−0.5	0.5	1	12.264	6.2	−2.7	−8.8	4.1	−1.2
	LS	1	0	0	0	−1	0	0	0	−1	−0.5	0.5	1	12.074	7.0	−4.0	−10.9	6.7	−1.2
	LS−HS													−0.190	0.8	−1.3	−2.1	2.6	0.0
	HS	1	0	0	0	−1	0	0	0	−1	0.5	0.5	1	12.264	6.2	−2.7	−8.8	4.1	−1.2
	LS	1	0	0	0	−1	0	0	0	−1	0.5	0.5	1	12.074	7.0	−4.0	−10.9	6.7	−1.2
	LS−HS													−0.190	0.8	−1.3	−2.1	2.6	0.0
	HS	1	0	0	0	−1	0	0	0	1	0	2	0.5	13.059	17.3	−4.5	−3.4	0.9	10.2
	LS	1	0	0	0	−1	0	0	0	1	0	2	0.5	12.881	22.8	−6.2	−4.4	2.6	14.8
	LS−HS													−0.178	5.5	−1.7	−1.0	1.7	4.6
	HS	1	0	0	0	−1	0	0	0	1	0	2	−0.5	13.059	17.3	−4.5	−3.4	0.9	10.2
	LS	1	0	0	0	−1	0	0	0	1	0	2	−0.5	12.881	22.8	−6.2	−4.4	2.6	14.8
	LS−HS													−0.178	5.5	−1.7	−1.0	1.7	4.6

ii. Co (ii)	State	Interaction Transformation Matrix and Vector												Centroid Distances	E_{Elec}	E_{Pol}	E_{Disp}	E_{Rep}	E_{Tot}
														(Å)			kJ mol^{-1}		
	HS	−1	0	0	0	−1	0	0	0	−1	1	1	1	7.223	−25.5	−14.2	−113.3	66.7	−86.3
	LS	−1	0	0	0	−1	0	0	0	−1	1	1	1	7.101	−31.3	−15.4	−123.1	80.4	−89.4
	LS−HS													−0.122	−5.8	−1.2	−9.8	13.7	−3.1
	HS	−1	0	0	0	−1	0	0	0	−1	0.5	0.5	1	8.567	−27.8	−12.3	−123.4	61.6	−101.9
	LS	−1	0	0	0	−1	0	0	0	−1	0.5	0.5	1	8.557	−27.2	−17.1	−128.2	71.0	−101.5
	LS−HS													−0.010	0.6	−4.8	−4.8	9.4	0.4
	HS	−1	0	0	0	−1	0	0	0	−1	0.5	1.5	1	10.884	1.5	−9.3	−82.5	36.4	−53.9
	LS	−1	0	0	0	−1	0	0	0	−1	0.5	1.5	1	10.921	−4.9	−8.3	−87.4	44.2	−56.3
	LS−HS													0.037	−6.4	1.0	−4.9	7.8	−2.4
	HS	−1	0	0	0	1	0	0	0	−1	0.5	0.5	0.5	10.562	−8.0	−3.4	−44.2	16.5	−39.1
	LS	−1	0	0	0	1	0	0	0	−1	0.5	0.5	0.5	10.555	−9.1	−3.8	−47.3	20.9	−39.5
														−0.007	−1.1	−0.4	−3.1	4.4	−0.4
	HS	−1	0	0	0	1	0	0	0	−1	0.5	−0.5	0.5	10.562	−8.0	−3.4	−44.2	16.5	−39.1
	LS	−1	0	0	0	1	0	0	0	−1	0.5	−0.5	0.5	10.555	−9.1	−3.8	−47.3	20.9	−39.5
	LS−HS													−0.007	−1.1	−0.4	−3.1	4.4	−0.4
	HS	1	0	0	0	−1	0	0	0	1	0	1	−0.5	9.640	−7.4	−3.1	−36.7	12.7	−34.5
	LS	1	0	0	0	−1	0	0	0	1	0	1	−0.5	9.672	−8.6	−3.7	−38.7	16.0	−35.0
	LS−HS													0.032	−1.2	−0.6	−2.0	3.3	−0.5
	HS	1	0	0	0	−1	0	0	0	1	0	1	0.5	9.640	−7.4	−3.1	−36.7	12.7	−34.5
	LS	1	0	0	0	−1	0	0	0	1	0	1	0.5	9.672	−8.6	−3.7	−38.7	16.0	−35
	LS−HS													0.032	−1.2	−0.6	−2.0	3.3	−0.5
	HS	−1	0	0	0	1	0	0	0	−1	1	0	0.5	13.12	−2.5	−2.0	−16.7	11.4	−9.9
	LS	−1	0	0	0	1	0	0	0	−1	1	0	0.5	12.992	−3.1	−2.8	−18.4	14.5	−9.7
														−0.128	−0.6	−0.8	−1.7	3.1	0.2
	HS	1	0	0	0	−1	0	0	0	1	−0.5	1.5	−0.5	14.094	−1.2	−1.8	−15.1	8.3	−9.8
	LS	1	0	0	0	−1	0	0	0	1	−0.5	1.5	−0.5	14.038	−2.6	−2.1	−16.7	11.3	−10.1
	LS−HS													−0.056	−1.4	−0.3	−1.6	3.0	−0.3
	HS	1	0	0	0	−1	0	0	0	1	0.5	1.5	0.5	14.094	−1.2	−1.8	−15.1	8.3	−9.8
	LS	1	0	0	0	−1	0	0	0	1	0.5	1.5	0.5	14.038	−2.6	−2.1	−16.7	11.3	−10.1

ii. Co (ii)	State	Interaction Transformation Matrix and Vector												Centroid Distances (Å)	E_{Elec}	E_{Pol}	E_{Disp} kJ mol^{-1}	E_{Rep}	E_{Tot}
	LS−HS													−0.056	−1.4	−0.3	−1.6	3.0	−0.3
	HS	1	0	0	0	−1	0	0	0	1	0.5	0.5	0.5	12.393	−2.6	−1.3	−17	4.7	−16.3
	LS	1	0	0	0	−1	0	0	0	1	0.5	0.5	0.5	12.290	−3.4	−1.7	−18.4	6.9	−16.6
	LS−HS													−0.103	−0.8	−0.4	−1.4	2.2	−0.3
	HS	1	0	0	0	−1	0	0	0	1	−0.5	0.5	−0.5	12.393	−2.6	−1.3	−17	4.7	−16.3
	LS	1	0	0	0	−1	0	0	0	1	−0.5	0.5	−0.5	12.290	−3.4	−1.7	−18.4	6.9	−16.6
														−0.103	−0.8	−0.4	−1.4	2.2	−0.3
	HS	−1	0	0	0	1	0	0	0	−1	1	0	1.5	10.548	4.1	−1.0	−14.1	1.8	−9.3
	LS	−1	0	0	0	1	0	0	0	−1	1	0	1.5	10.587	2.3	−1.0	−14.6	2.8	−10.5
	LS−HS													0.039	−1.8	0.0	−0.5	1.0	−1.2
	HS	1	0	0	0	1	0	0	0	1	0.5	0.5	0	13.693	1.1	−0.2	−5.0	0.4	−3.6
	LS	1	0	0	0	1	0	0	0	1	0.5	0.5	0	13.554	0.7	−0.3	−5.3	0.5	−4.4
	LS−HS													−0.139	−0.4	−0.1	−0.3	0.1	−0.8
	HS	1	0	0	0	1	0	0	0	1	−0.5	−0.5	0	13.693	1.1	−0.2	−5.0	0.4	−3.6
	LS	1	0	0	0	1	0	0	0	1	−0.5	−0.5	0	13.554	0.7	−0.3	−5.3	0.5	−4.4
	LS−HS													−0.139	−0.4	−0.1	−0.3	0.1	−0.8

**Iii. Tet-Phen**	**State**	**Interaction Transformation Matrix and Vector**												**Centroid Distances**	$\boldsymbol{E}_{\text{Elec}}$	$\boldsymbol{E}_{\text{Pol}}$	$\boldsymbol{E}_{\text{Disp}}$	$\boldsymbol{E}_{\text{Rep}}$	$\boldsymbol{E}_{\text{Tot}}$
														(Å)			kJ mol^{-1}		
	HS	1	0	0	0	−1	0	0	0	1	0	−0.5	−0.5	8.402	−26.7	−22.5	−78.9	51.6	−76.6
	LS	1	0	0	0	−1	0	0	0	1	0	−0.5	−0.5	8.369	−29.9	−24	−74.3	52.3	−75.9
	LS−HS													−0.033	−3.2	−1.5	4.6	0.7	0.7
	HS	1	0	0	0	−1	0	0	0	1	0	−0.5	0.5	8.402	−26.7	−22.5	−78.9	51.5	−76.6
	LS	1	0	0	0	−1	0	0	0	1	0	−0.5	0.5	8.369	−29.9	−24	−74.3	52.3	−75.9
	LS−HS													−0.033	−3.2	−1.5	4.6	0.8	0.7
	HS	1	0	0	0	−1	0	0	0	1	0	0.5	−0.5	8.402	−26.7	−22.5	−78.9	51.5	−76.6
	LS	1	0	0	0	−1	0	0	0	1	0	0.5	−0.5	8.369	−29.3	−23.8	−73.6	52.8	−73.9
	LS−HS													−0.033	−2.6	−1.3	5.3	1.3	2.7
	HS	1	0	0	0	−1	0	0	0	1	0	0.5	0.5	8.402	−26.7	−22.5	−78.9	51.4	−76.6
	LS	1	0	0	0	−1	0	0	0	1	0	0.5	0.5	8.369	−29.3	−23.8	−73.6	52.8	−73.9
														−0.033	−2.6	−1.3	5.3	1.4	2.7
	HS	−1	0	0	0	−1	0	0	0	−1	0	0	1	8.583	−46.5	−18.4	−57.0	28.0	−94.0
	LS	−1	0	0	0	−1	0	0	0	−1	0	0	1	8.330	−49.1	−20.6	−67.2	38.1	−98.8
	LS−HS													−0.253	−2.6	−2.2	−10.2	10.1	−4.8
	HS	−1	0	0	0	−1	0	0	0	−1	1	0	1	8.583	−46.5	−18.4	−57.0	28.0	−94.0
	LS	−1	0	0	0	−1	0	0	0	−1	1	0	1	8.330	−48.6	−20.6	−67.1	38.2	−98.1
	LS−HS													−0.253	−2.1	−2.2	−10.1	10.2	−4.1
	HS	−1	0	0	0	1	0	0	0	−1	0	0.5	0.5	11.559	−27.4	−6.5	−8.5	3.2	−39.2
	LS	−1	0	0	0	1	0	0	0	−1	0	0.5	0.5	11.311	−28.4	−6.0	−8.0	2.6	−39.9
	LS−HS													−0.248	−1.0	0.5	0.5	−0.6	−0.7
	HS	−1	0	0	0	1	0	0	0	−1	0	−0.5	0.5	11.559	−27.4	−6.5	−8.5	3.2	−39.2
	LS	−1	0	0	0	1	0	0	0	−1	0	−0.5	0.5	11.311	−28.4	−6.0	−8.0	2.6	−39.9
														−0.248	−1.0	0.5	0.5	−0.6	−0.7
	HS	−1	0	0	0	1	0	0	0	−1	1	−0.5	0.5	11.559	−27.4	−6.5	−8.5	3.2	−39.1
	LS	−1	0	0	0	1	0	0	0	−1	1	−0.5	0.5	11.311	−28.3	−6.1	−8.1	2.6	−40.0
	LS−HS													−0.248	−0.9	0.4	0.4	−0.6	−0.9
	HS	−1	0	0	0	1	0	0	0	−1	1	0.5	0.5	11.559	−27.4	−6.5	−8.5	3.2	−39.1
	LS	−1	0	0	0	1	0	0	0	−1	1	0.5	0.5	11.311	−28.3	−6.1	−8.1	2.6	−40.0

Iii. Tet-Phen	State	Interaction Transformation Matrix and Vector												Centroid Distances (Å)	E_{Elec}	E_{Pol}	E_{Disp}	E_{Rep}	E_{Tot}
																	kJ mol^{-1}		
	LS−HS													−0.248	−0.9	0.4	0.4	−0.6	−0.9
	HS	1	0	0	0	1	0	0	0	1	0	0	−1	9.226	−30.7	−8.7	−13.1	2.9	−49.7
	LS	1	0	0	0	1	0	0	0	1	0	0	−1	9.372	−30.5	−9.2	−13.3	3.2	−49.7
	LS−HS													0.146	0.2	−0.5	−0.2	0.3	0.0
	HS	1	0	0	0	1	0	0	0	1	0	0	1	9.226	−30.7	−8.7	−13.1	2.9	−49.7
	LS	1	0	0	0	1	0	0	0	1	0	0	1	9.372	−30.5	−9.2	−13.3	3.2	−49.7
														0.146	0.2	−0.5	−0.2	0.3	0.0
	HS	−1	0	0	0	1	0	0	0	−1	0	−0.5	1.5	12.447	6.1	−1.8	−6.9	1.2	−1.5
	LS	−1	0	0	0	1	0	0	0	−1	0	−0.5	1.5	12.284	6.5	−1.8	−7.2	1.3	−1.3
	LS−HS													−0.163	0.4	0.0	−0.3	0.1	0.2
	HS	−1	0	0	0	1	0	0	0	−1	0	0.5	1.5	12.447	6.1	−1.8	−6.9	1.2	−1.5
	LS	−1	0	0	0	1	0	0	0	−1	0	0.5	1.5	12.284	6.5	−1.8	−7.2	1.2	−1.3
	LS−HS													−0.163	0.4	0.0	−0.3	0.0	0.2
	HS	−1	0	0	0	1	0	0	0	−1	1	−0.5	1.5	12.447	6.1	−1.8	−6.9	1.1	−1.5
	LS	−1	0	0	0	1	0	0	0	−1	1	−0.5	1.5	12.284	6.5	−1.8	−7.2	1.3	−1.3
	LS−HS													−0.163	0.4	0.0	−0.3	0.2	0.2

(1) Bryant, M. J.; Maloney, A. G. P.; Sykes, R. A. Predicting mechanical properties of crystalline materials through topological analysis. *CrystEngComm* 2018, *20* (19), 2698.
(2) Guionneau, P.; Marchivie, M.; Bravic, G.; Letard, J.-F.; Chasseau, D. Structural Aspects of Spin Crossover — Example of the [FeIILn(NCS)2] Complexes. *Top. Curr. Chem.* 2004, *234*, 97.
